# Sobralene, a new sex-aggregation pheromone and likely shunt metabolite of the taxadiene synthase cascade, produced by a member of the sand fly *Lutzomyia longipalpis* species complex

**DOI:** 10.1016/j.tetlet.2018.03.088

**Published:** 2018-05-16

**Authors:** Matthew J. Palframan, Krishna K. Bandi, James G.C. Hamilton, Gerald Pattenden

**Affiliations:** a School of Chemistry, The University of Nottingham, University Park, Nottingham NG7 2RD, UK; b Division of Biomedical and Life Sciences, Faculty of Health and Medicine, Lancaster University, Lancaster LA1 4YG, UK

**Keywords:** Verticillenes, Sand fly, Taxadiene synthase, Insect pheromone, *Lutzomyia longipalpis*

## Abstract

A new sex-aggregation pheromone, sobralene, produced by the sand fly *Lutzomyia longipalpis* from Sobral (Ceará State, Brazil) is shown to have the novel 6,12-membered ring-fused diterpene structure **3.** It is proposed that sobralene is a likely shunt metabolite of the taxadiene synthase-catalysed cyclisation of geranygeranyl diphosphate.

## Highlights

• Established the structure of a diterpenoid sex-aggregation pheromone (sobralene).
• Sobralene has a novel bicyclic ring, related to the verticillenes.
• Propose sobralene has its origins as a shunt metabolite of taxadiene synthase.

## Graphical abstract

**Figure:**
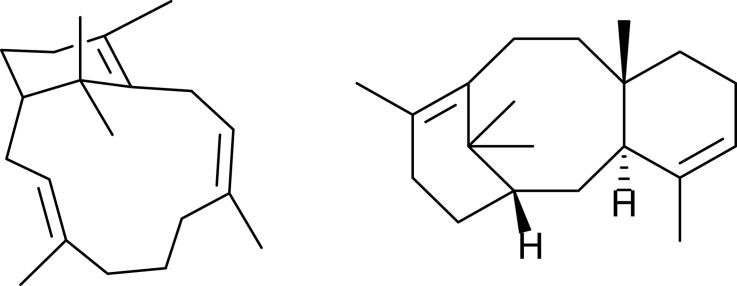

The sand fly species complex *Lutzomyia longipalpis* is the main carrier of the Protist parasite *Leishmania infantum,* the causative agent of American visceral leishmaniasis (AVL), a potentially fatal human disease in South and Central America.^1^, ^2^ The males of the species complex produce different sex-aggregation pheromones depending on which member they are, and these chemicals have been studied for both their taxonomic and vector control potential.^3^, ^4^, ^5^ Earlier, two pheromones, from populations from Lapinha Cave (Minas Gerais State) and Jacobina (Bahia State) in Brazil, were shown to be the homosesquiterpenes (*S*)-9-methylgermacrene-B (**1**) and 3-methyl-α-himachalene **2**, respectively (Fig. 1).^6^, ^7^, ^8^, ^9^ For several years other pheromones, from populations from Jaíbas (Minas Gerais State) and Sobral (Ceará State, 2S population),^10^ have been thought to have cembrene (14-membered ring) structures on the basis of GCMS data.^10^, ^11^

**Fig. 1:**
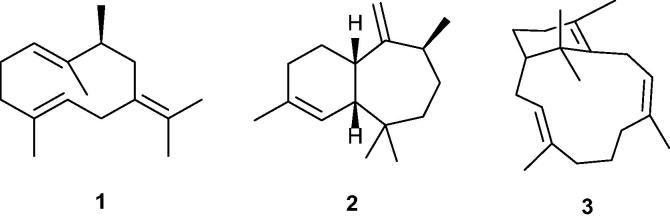
Sex-aggregation pheromones produced by the sand fly species complex *L. longipalpis.*

We have now examined the NMR spectroscopic data for the sex-aggregation pheromone produced by the 2-spot *L. longipalpis* population from Sobral, Brazil. These data establish that the pheromone has the novel bicyclo [9.3.1] pentadeca-*E*-3,4-*Z*-8,9-triene structure **3**. Herein, we discuss the essential NMR spectroscopic, and other, data which led to the assignment of structure **3**, and its likely biosynthesis in the sand fly.

Secretion from approximately two thousand male Sobral-2S sand flies, held in Lancaster University, was collected in hexane over several months during 2017. GLC analysis of the extract showed the presence of one major (>90%) and two minor (*ca.* 7% and 2%) components and GC–MS analysis demonstrated that each component in the mixture was a diterpenoid hydrocarbon. The major component in the extract showed a weak HR-EI molecular ion at *m*/*z* 272.2501, corresponding to a molecular formula C_20_H_32_ and indicating five degrees of unsaturation; the major fragmentation peak at *m*/*z* 257 corresponded to the loss of CH_3_ from the molecular ion.

The hexane extract was stored in the dark in a freezer at −20 °C and then carefully evaporated to dryness using a stream of dry argon gas at room temperature. The residue, estimated to be approximately 100 µg, was then taken up in benzene‑*d_6_* for analysis by NMR spectroscopy. The ^13^C NMR spectrum, alongside 2D HSQC and HMBC spectra, showed signals corresponding to four olefinic quaternary carbons (δ 137.0, 135.3, 131.5, 127.4), two olefinic methine carbons (δ 125.4, 124.1), one aliphatic methine carbon (δ 43.4), seven aliphatic methylene carbons (δ 33.7, 38.1, 24.2, 2 × 28.5, 31.2, 27.6), five methyl carbons (δ 33.0, 25.4, 23.4, 22.4, 16.7), and one aliphatic quaternary carbon (δ 37.6) (Table 1).

**Table 1:** ^1^H and ^13^C NMR spectroscopic data for sobralene **3**.

**Figure:**
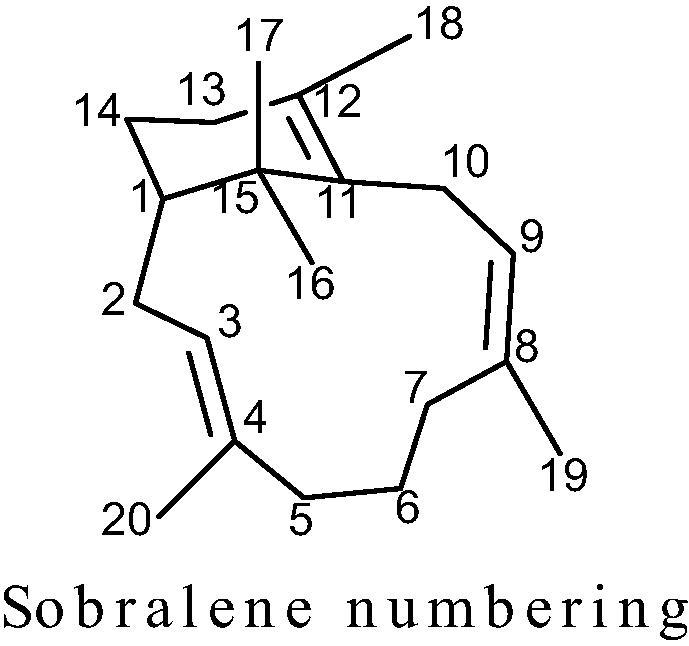

Carbon	δ_C_ (126 MHz)	Proton	δ_H_ (800 MHz) (*J* in Hz)
1	43.4, CH	1	1.57 (m)
2	33.7, CH_2_	2a	2.68 (ddd, *J* = 16.4, 10.4, 6.5)
		2b	2.02 (m)
3	125.4, CH	3	5.43 (dd, *J* = 10.4, 4.0)
4	131.5, C	4	
5	38.1, CH_2_	5a	1.90 (m)
		5b	1.90 (m)
6	24.2, CH_2_	6a	1.54 (m)
		6b	1.41 (m)
7	28.5, CH_2_	7a	2.34 (ddd, *J* = 13.2, 8.8, 3.9)
		7b	2.02 (m)
8	135.3, C	8	
9	124.1 CH	9	5.40 (app. t, *J* = 6.6)
10	28.5, CH_2_	10a	2.85 (dm, *J* = 16.7)
		10b	2.80 (dd, *J* = 16.7, 7.1)
11	137.0, C	11	
12	127.4, C	12	
13	31.2 CH_2_	13a	2.18 (m)
		13b	1.87 (m)
14	27.6, CH_2_	14a	2.18 (m)
		14b	1.52 (m)
15	37.6, C	15	
16	25.4, CH_3_	16	1.15 (s)
17	33.0, CH_3_	17	1.08 (s)
18	22.4, CH_3_	18	1.64 (s)
19	23.4, CH_3_	19	1.70 (s)
20	16.7, CH_3_	20	1.56 (s)

The ^1^H NMR and HSQC spectra revealed the presence of two olefinic protons (δ 5.43 (dd) and 5.40 (app. t)); one aliphatic methine proton (δ 1.57); fourteen diastereotopic methylene protons (δ 1.75 – 3.0); three vinyl methyl singlets (δ 1.70, 1.64 and 1.56); and two aliphatic methyl group singlets (δ 1.15 and 1.08) (Table 1).

The above NMR spectroscopic data accounted for three degrees of unsaturation, with the remaining two degrees of unsaturation accounted for by the presence of a C_20_-bicyclic carbon skeleton in the sobralene structure. The proton and carbon connectivities in structure **3** were determined following analysis of its COSY, TOSCY and HBMC spectra (Fig. 2). Thus, COSY correlations were observed between H-3 and H-2a, H-2b and H-20; between H-1 and H-2a and H-2b; between H-9 and H10a/H10b and H-19; and between H18 and H-13. Analysis of the HMBC spectrum for **3** revealed key correlations between C-1 and H-2a, H-2b, H-14, H-16 and H-17; between H-3 and C-2, C-5, and C-20; and between H-9 and C-10, C-11, and C-19.

**Fig. 2:**
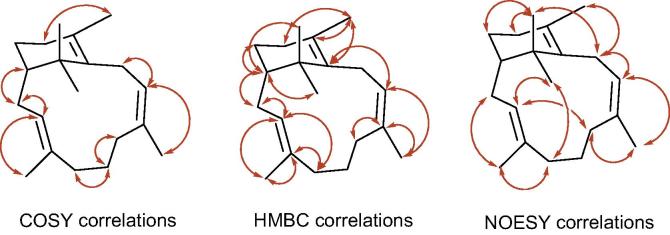
Key COSY, HMBC and NOESY correlations of sobralene **3**.

The configuration of the two tri-substituted alkene bonds in the 12-membered ring of sobralene **3** were determined from NOESY correlations (Fig. 2). Thus the NOESY correlations between H-20 and H-2a, H-5 and H-16; between H-3 and H-2b, H-7a and H-16, along with the absence of a NOESY correlation between H-3 and H-20 indicated that the C3, C4 alkene bond in **3** had an *E-*configuration. Correspondingly, the NOESY correlations between H-9 and H-10 and H-19; between H19 and H-7a, H-7b and H9 indicated that the alkene bond between C8 and C9 had the *Z-*configuration. The observed NOESY correlations also supported the overall structure assigned to sobralene through its COSY, TOSY and HMBC spectra.

Although “verticillene”-type diterpene hydrocarbons have been isolated from plants^12^, ^13^, ^14^, ^15^ and liverworts^16^ to the best of our knowledge, this is the first time that one has been reported in insects. The verticillyl carbocation **5** is a central intermediate in the cascade of cyclisations from geranygeranyl diphosphate (GGPP) **4** which lead to the tricyclic ring system in taxadiene **8** and in oxygen-functionalised derivatives of **8** such as the antitumor agent taxol.^17^ A crucial step in the taxadiene synthase (TXS) cascade is the transfer of a proton from C11 to C7 in **5** leading to the corresponding C8 verticillyl carbocation **10**, which initiates the cyclisation producing the six-membered C-ring in taxadiene **8** (Scheme 1).17a However, it is generally recognised that the transfer of the proton from C11 to C7 in **5** is a two-step process, involving i) initial transfer of the proton at C11 to C3 in **5** leading to the C4-verticillyl carbocation **6**, followed by ii) transfer of the proton at C3 to C7 in **6** producing **7**.(b), (c) Significantly, studies have also revealed that a change in conformation of the C8 carbocation **7**, *i.e.* from conformation **7** to conformation **10**, is necessary in order to facilitate its cyclisation to the taxadiene carbocation intermediate **9** en route to taxadiene **8**.(c), 18

**Scheme 1:**
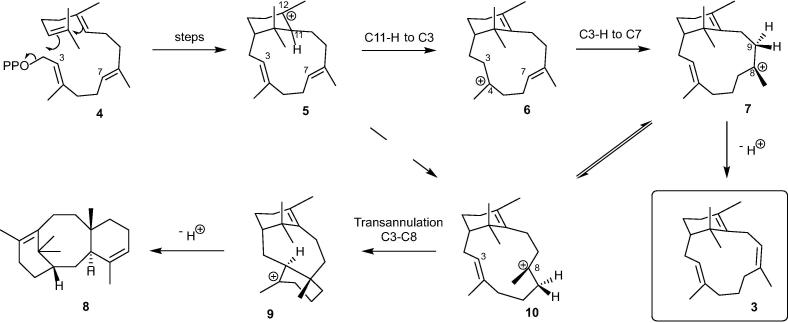
Outline of the taxadiene synthase (TXS) cascade, and proposal for the origin of sobralene **3** in *L. longipalpis.*

It seems likely that sobralene **3** is a shunt metabolite of the above TXS cascade. Thus, in the absence of a change in the conformation of the C8 verticillyl carbocation **7**, the intermediate could instead simply eliminate its α-proton at C9 thereby producing the C8,9-alkene bond in sobralene with the *Z*-configuration. We secured some support for this proposal when we examined the structure of one of the minor constituents in the secretion from *L. longipalpis.* Thus, the minor constituent, which eluted at 30.3 min. in GC, in the secretion [*cf*. sobralene 29.95 min.] did not separate from authentic taxadiene **8** in mixed GC analysis. Furthermore, its mass spectrum fragmentation pattern following GCMS analysis was superimposable on that recorded for authentic taxadiene. The second minor constituent in the secretion, eluting at 30.7 min, had a mass spectrum fragmentation pattern which supported a verticillene-type structure, but we were not able to establish its likely structure unambiguously.

The presence of taxadiene **8** as a minor constituent in the secretion from the sand fly lends support to our suggestion that sobralene is most likely a shunt metabolite of the taxadiene synthase (TXS) cascade. Interestingly,other researchers have very recently carried out some targeted engineering of TXS which has resulted in the production of various isomeric verticillenes of relevance to the biosynthesis of taxadiene.^19^ The accumulation of taxadiene in *L. longipalpis* could be the result of transannular cyclisation from small amounts of conformation **10** in the C8 carbocation intermediate **7** (Scheme 1). It could also result from isomerisation followed by cyclisation of sobralene **3** in the sand fly or during its isolation and/or storage. Future work is now in place to unravel these intricacies and provide a clearer picture of the origin of sobralene **3** and its biological relationships with taxadiene **8** and any additional verticillene-type metabolites which might be present in *L. longipalpis.*
