# Supplementary material for: Sobralene, a new sex-aggregation pheromone and likely shunt metabolite of the taxadiene synthase cascade, produced by a member of the sand fly Lutzomyia longipalpis species complex
Source: Tetrahedron Lett. 2018 May 16;59(20):1921–3. doi: 10.1016/j.tetlet.2018.03.088 (PMC5937913; doi:10.1016/j.tetlet.2018.03.088)

ESI Data

Sobralene, a new sex pheromone of the male sandfly *Lutzomia longipalpis.* A plausible shunt metabolite in the taxadiene synthase cascade

Krishna K. Bandi,[a] James G. C.Hamilton,[a] Matthew J. Palframan,[b] and Gerald Pattenden*[b]

General Experimental Procedures.

i) A colony of diterpene producing Sobral-2S *L. longipalpis* sandflies was initially established by isofemale rearing in 2013 from males and females collected in Sobral, Ceará State (3˚ 41' S, 40˚ 20' W) (Lawyer et al., 2017). Each week surplus males (100-200, > 5 day old) were harvested from the colony after gravid females had been removed for egg laying and placed in a clean conical flask (250 ml) containing hexane (50 ml; Merck SupraSolv grade) and stored (-20 ^{o}C).[1] The sample of sobralene used for structural determination was prepared from ca. 1,500-2,000 males collected between May 2017 and September 2017. The hexane extract was first purified by passing through filter paper (to remove the insect material) and then passed through a Pasteur-pipette silica-gel column (0.5 g, Normasil, VWR Ltd activated by heating at 100 °C) to remove non-hydrocarbon compounds. The hexane extract was stored in the dark in a freezer at -20° C and then carefully evaporated to dryness using a stream of dry argon gas at room temperature. The residue, estimated to be approx. 100 micrograms, was then taken up in d_6 benzene for analysis by NMR spectroscopy.

ii) NMR spectra were recorded using a Bruker AV(III)500 spectrometer equipped with cryoprobe operating at frequencies of 500 MHz (^{1}H) and 126 MHz (^{13}C) and Bruker AV(III)800 spectrometer equipped with inverse cryoprobe operating at frequencies of 800 MHz (^{1}H) and 200 MHz (^{13}C).[2] The spectra were measured in C_6D_6, with the ^{1}H and ^{13}C NMR chemical shifts (δ) expressed in ppm with reference to the solvent signals [C_6D_6: δ_H 7.16 and δ_C 128.0]. Coupling constants are given in Hz.

iii) GCMS spectra were recorded using a JEOL AccuTOF GCX (JEOL Ltd., Tokyo, Japan) Mass Spectrometer with a Agilent 7890B (Agilent Technologies Inc, Wilmington, USA) Gas Chromatograph.[3] The column was a 30.0 m × 0.25 mm i.d., 0.20 uM film thickness Thermo TG-POLAR ThermoFisher Scientific Corp (Waltham, Mass, USA). The gas chromatograph condition were: initial oven temperature 40 °C which was held for 3.0 min, followed by a 5 °C per min up to 260 °C

[1] We are grateful to Dr Dan Bray for help in establishing the Sobral 2S colony, and to Pam Taylor, Ann Underhill and Raquel Juliana Vionette do Amaral for maintaining the sand fly colony in Lancaster University. We also thank Fraser Mitchell for analytical support in Lancaster.

[2] We thank Kevin Butler and Huw Williams for their excellent collaboration with the NMR spectroscopy investigations in Nottingham.

[3] We thank Mick Cooper for his help with the gas chromatograph mass spectrometry investigations in Nottingham.

GCMS of sample of Sobralene – GS data GCMS of sample of Sobralene.

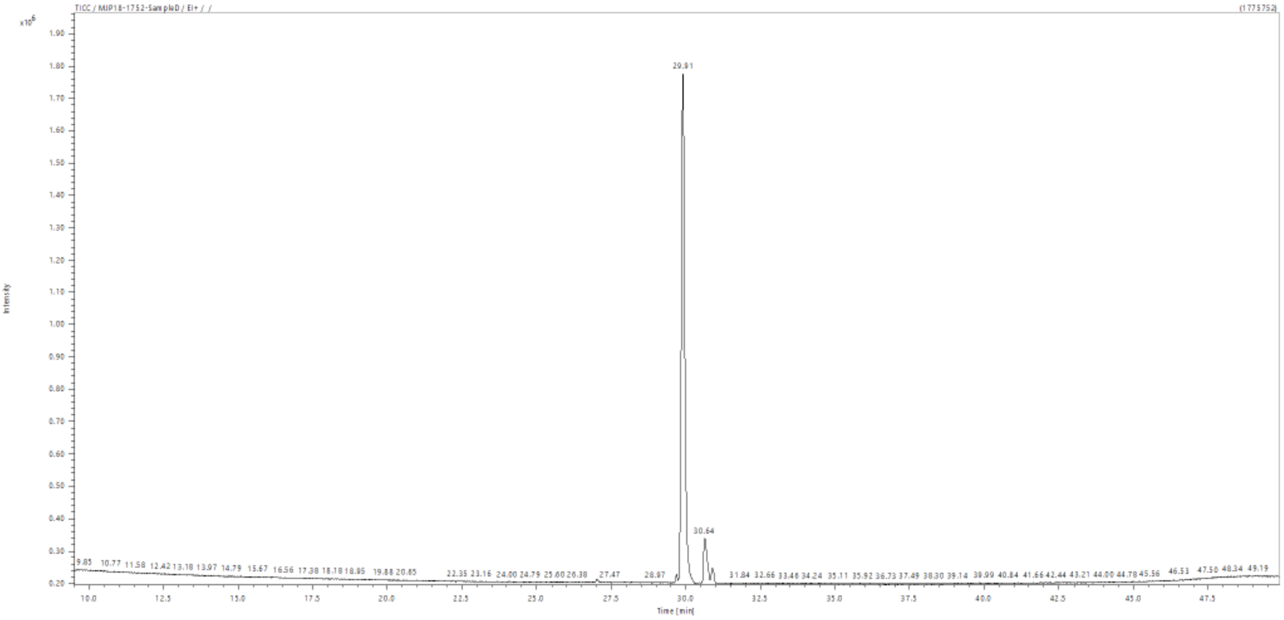

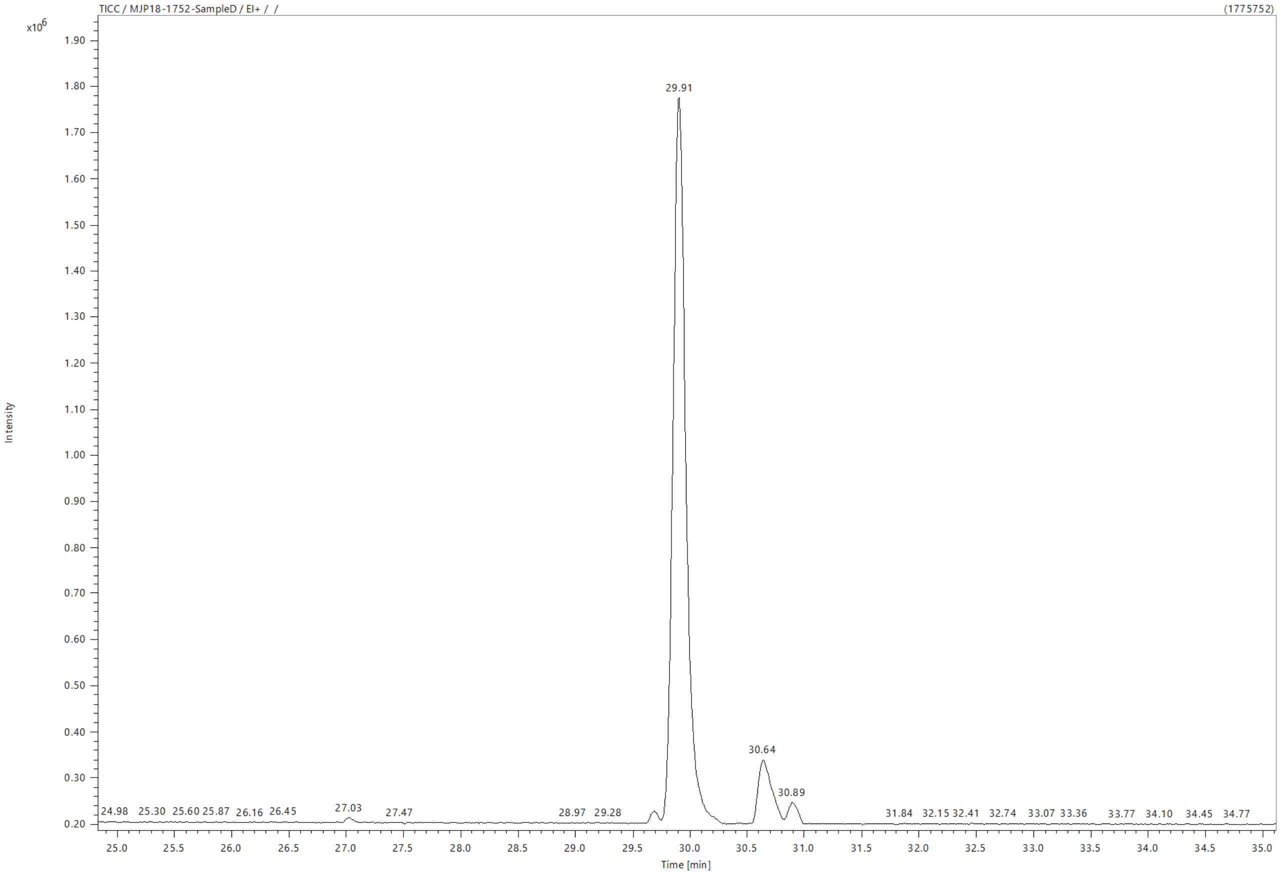

GCMS of sample of Sobralene – Mass spec data for major peak 29.91 min.

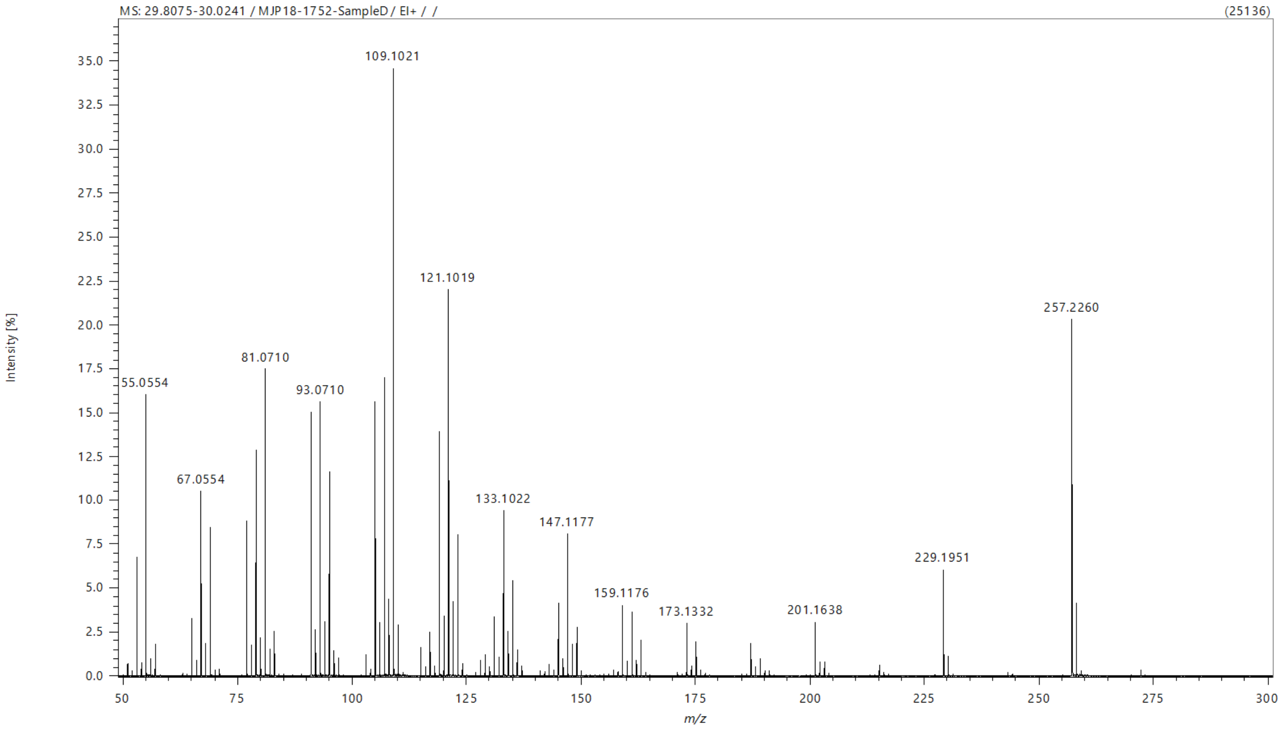

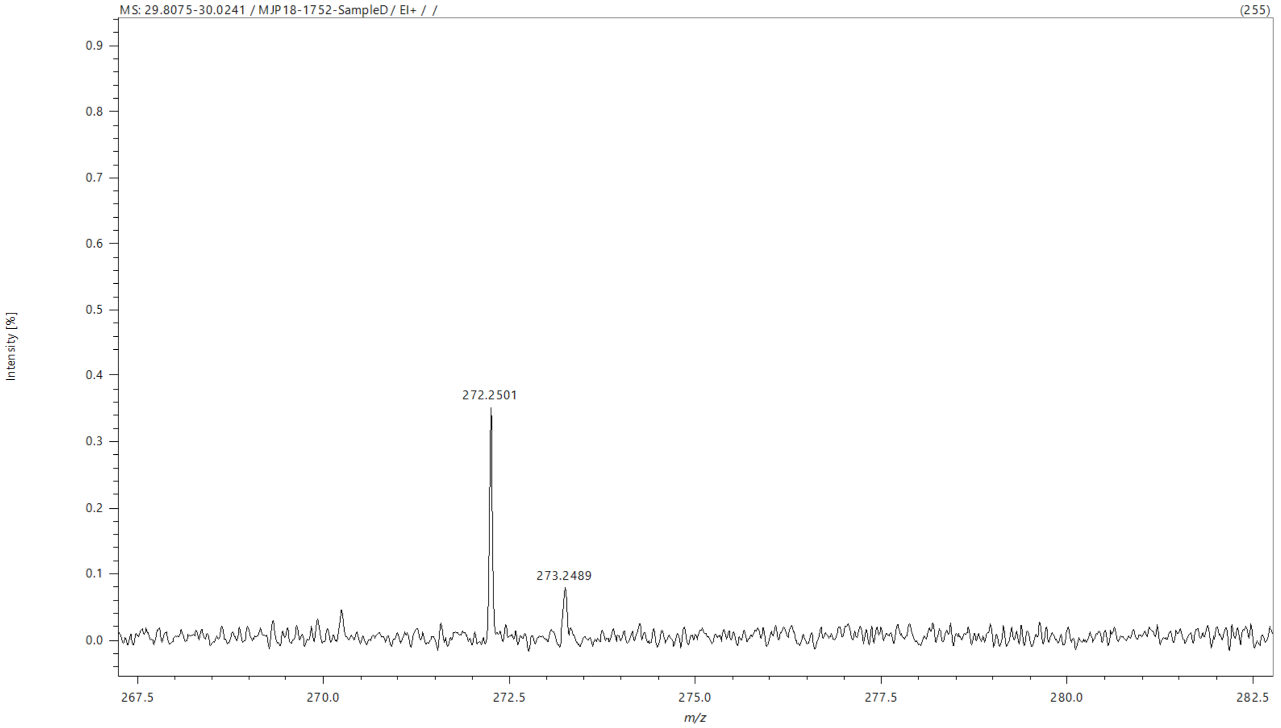

Sobralene ($C_{20}H_{32}^{+}$, M^{+}) requires 272.2499; HR-EI-*m/z* found 272.2501,

NMR data for Sobralene – 800 MHz

1H

TOSCY

HSQC

HMBC

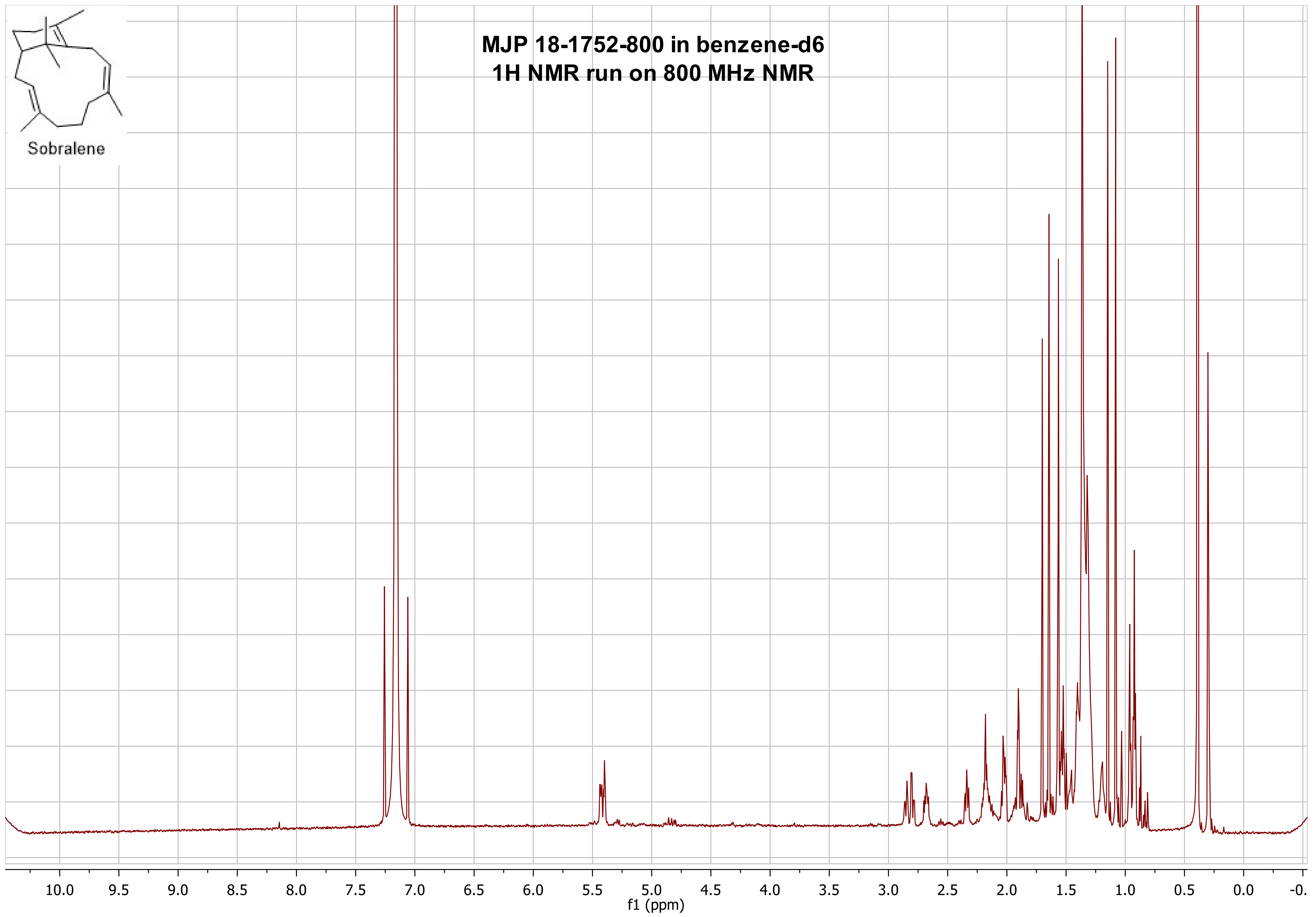

MJP 18-1752-800 in benzene-d6
1H NMR run on 800 MHz NMR
Sobralene
10.0
9.5
9.0
8.5
8.0
7.5
7.0
6.5
6.0
5.5
5.0
4.5
4.0
3.5
3.0
2.5
2.0
1.5
1.0
0.5
0.0
-0.
f1 (ppm)

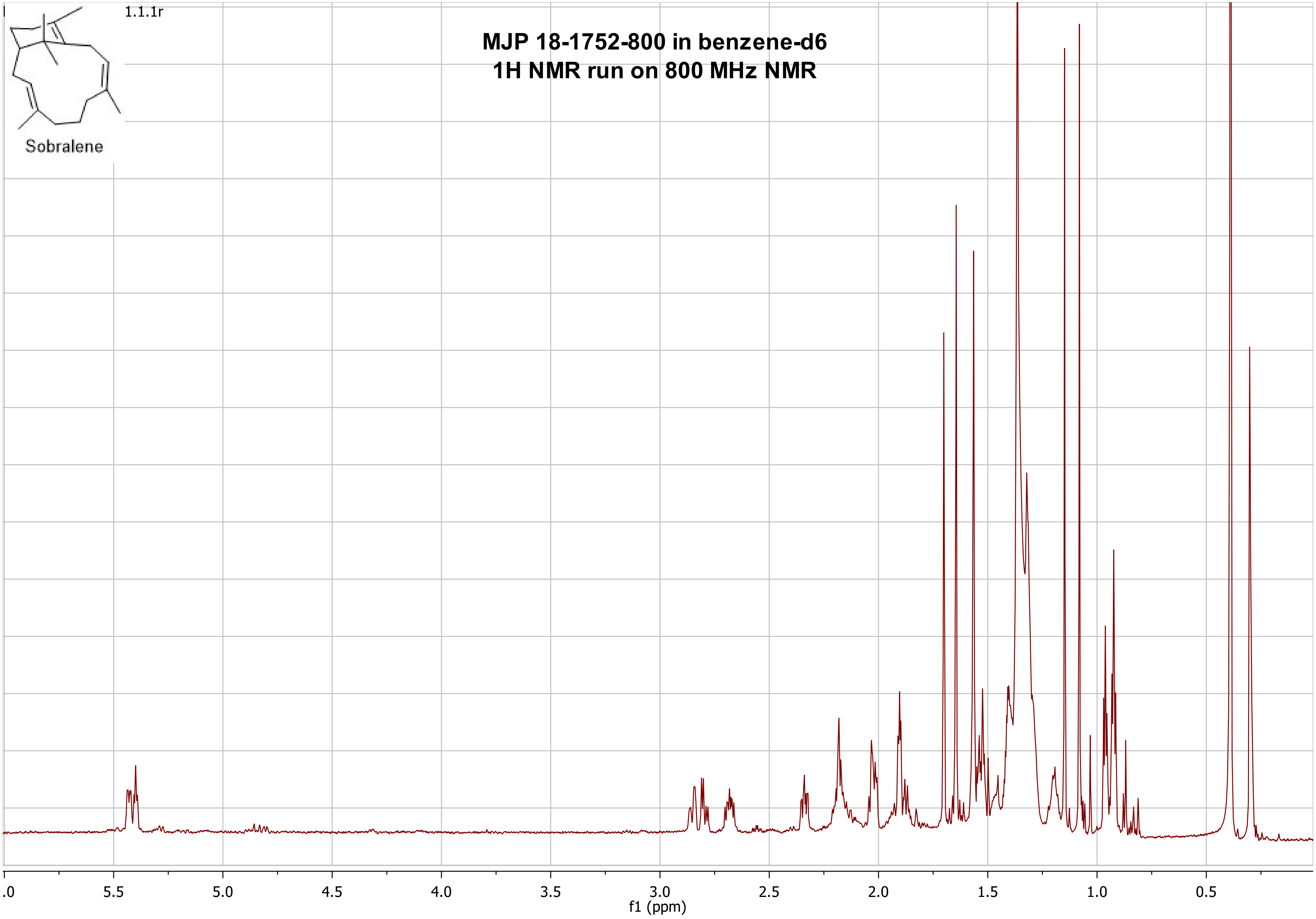
1.1.1r
MJP 18-1752-800 in benzene-d6
1H NMR run on 800 MHz NMR
Sobralene
.0
5.5
5.0
4.5
4.0
3.5
3.0
2.5
2.0
1.5
1.0
0.5
f1 (ppm)

MJP 18-1752-800 in benzene-d6
Dept editted HSQC NMR run on 800 MHz NMR

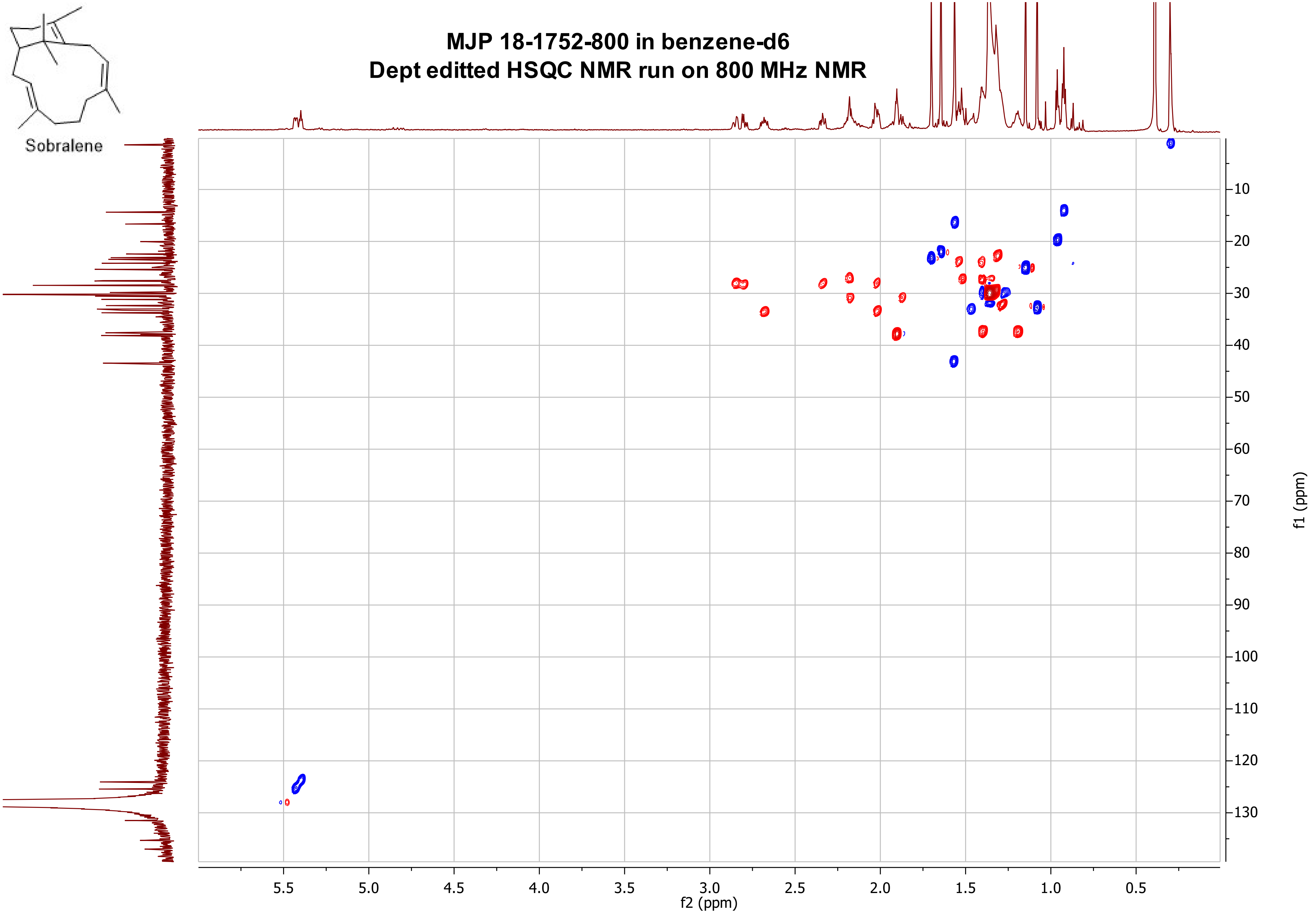

MJP 18-1752-800 in benzene-d6
HMBC NMR run on 800 MHz NMR

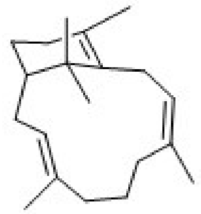
Sobralene

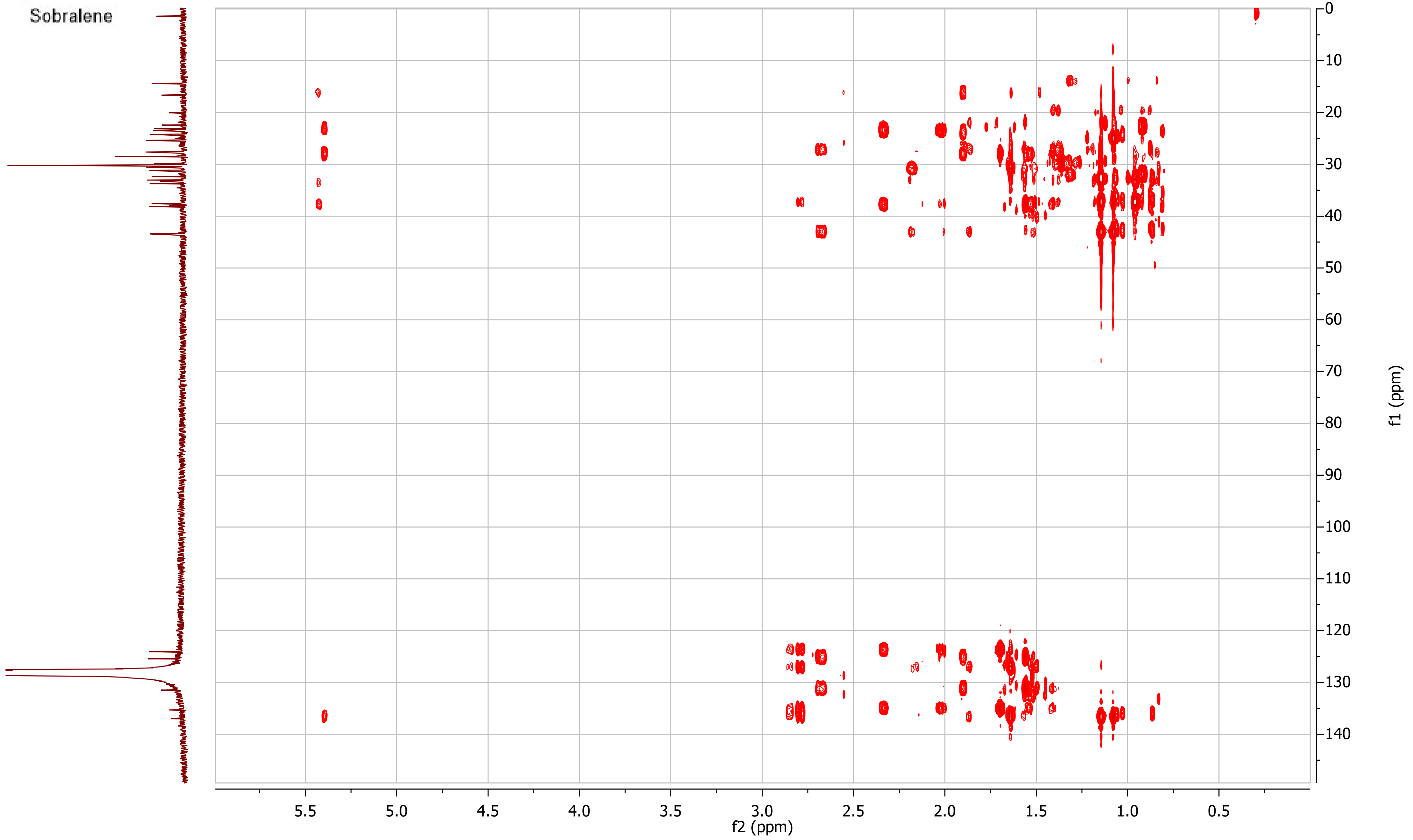

MJP 18-1752-800 in benzene-d6
TOSCY NMR run on 800 MHz NMR

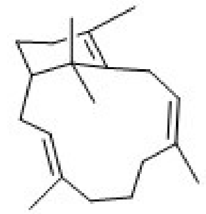

Sobralene

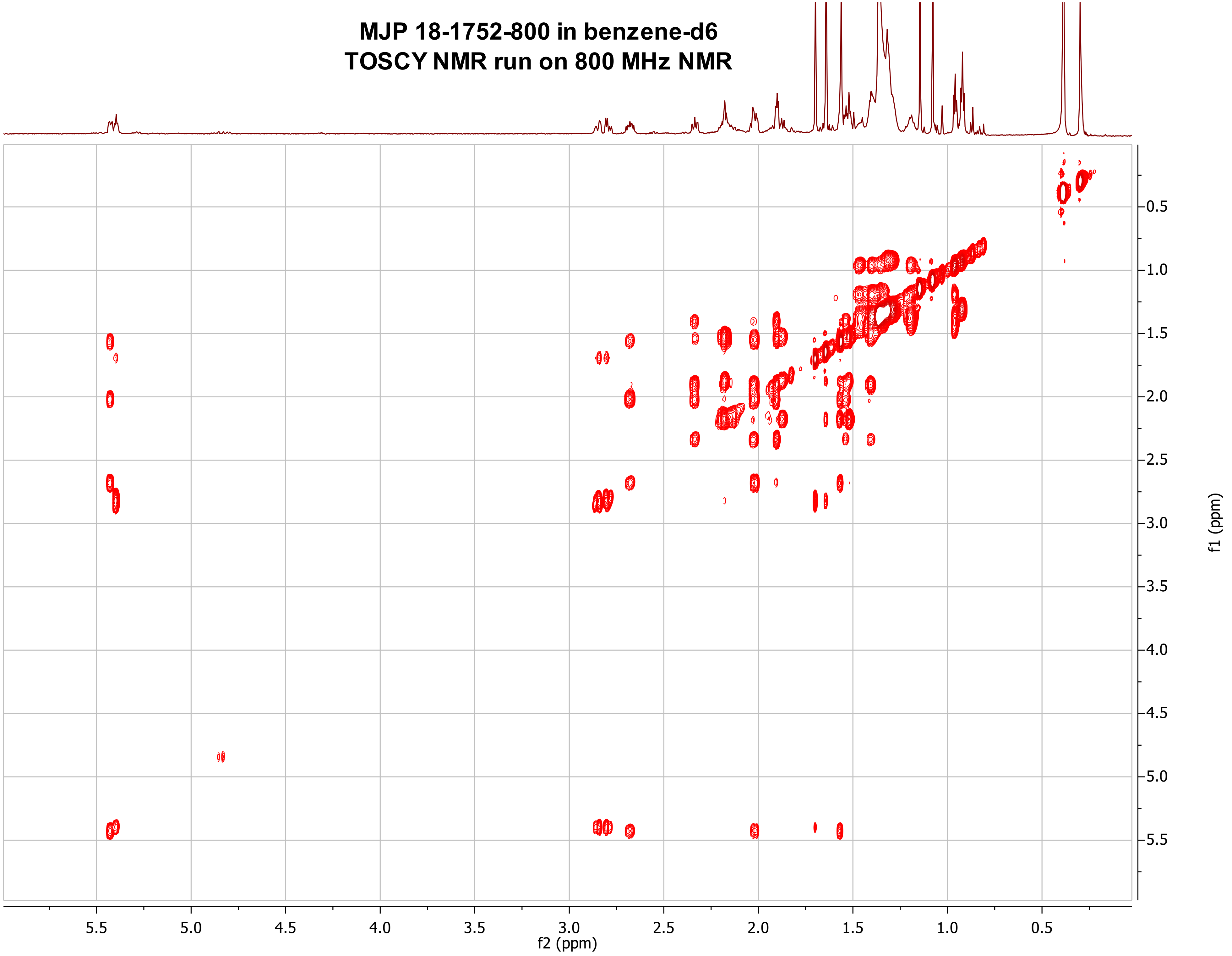

NMR data for Sobralene – 500 MHz

1H

COSY

HSQC

HMBC

NOESY

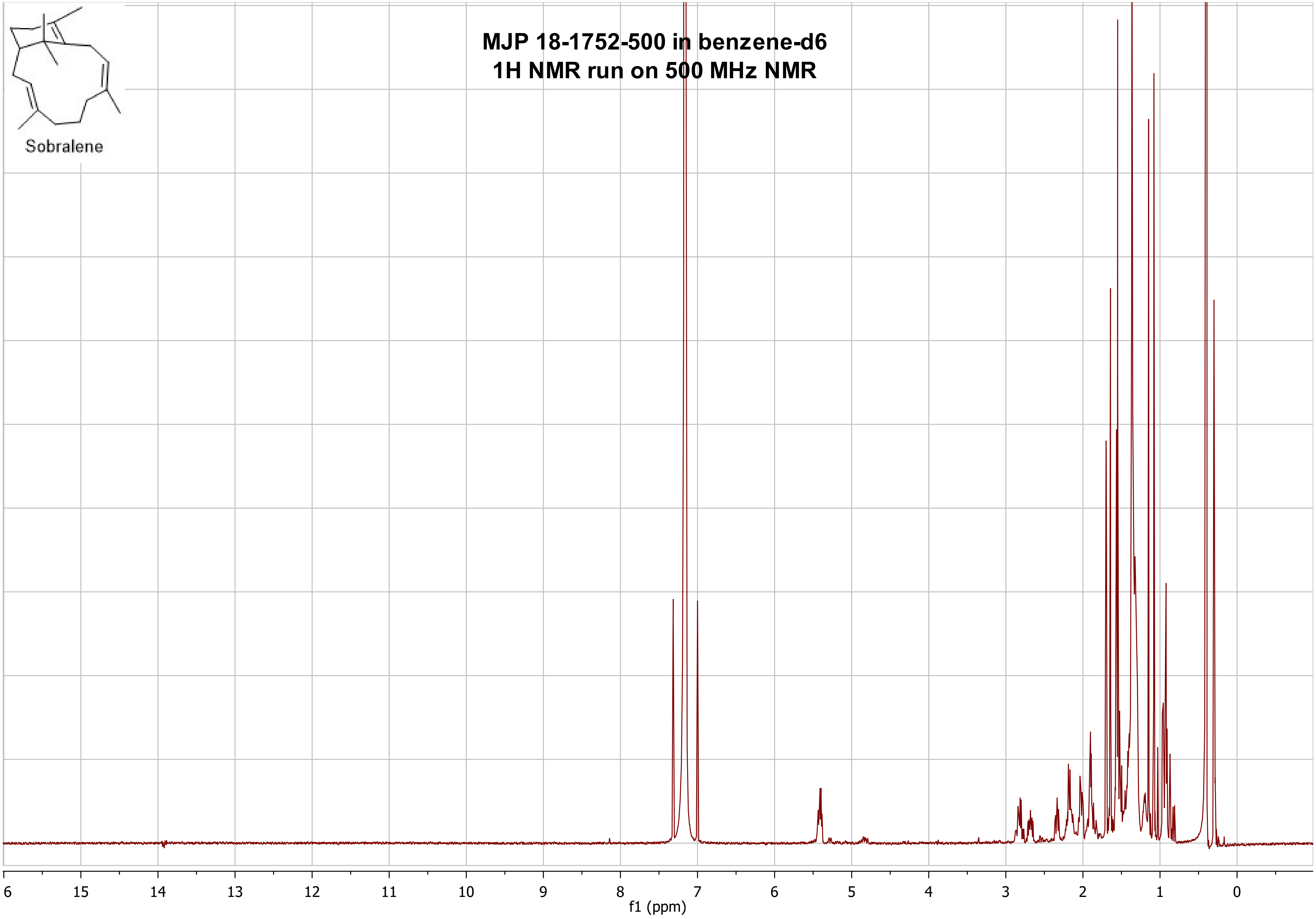

MJP 18-1752-500 in benzene-d6
1H NMR run on 500 MHz NMR
Sobralene
6
15
14
13
12
11
10
9
8
7
6
5
4
3
2
1
0
f1 (ppm)

MJP 18-1752-500 in benzene-d6
1H NMR run on 500 MHz NMR

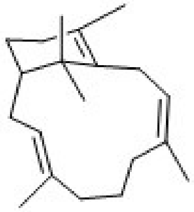

Sobralene

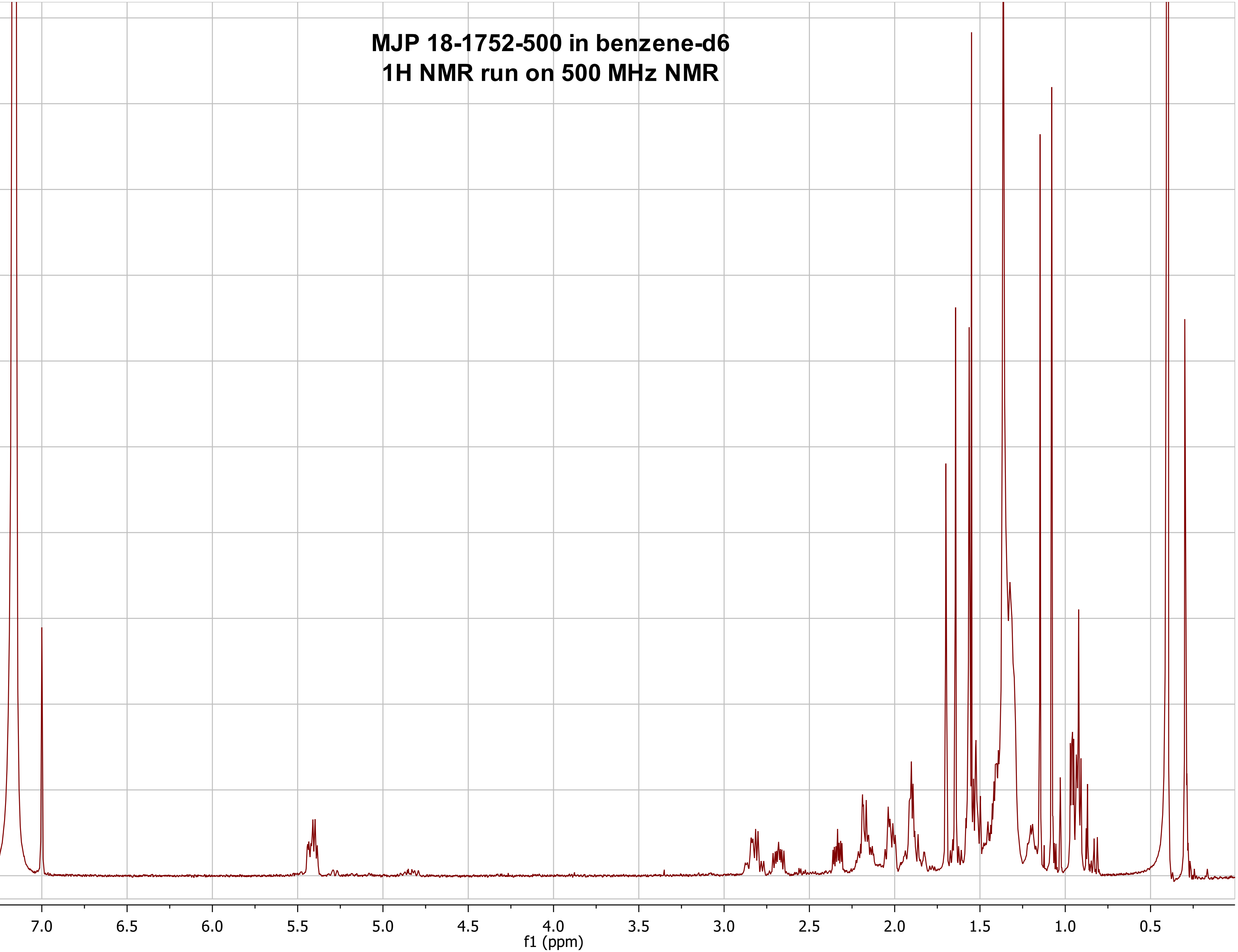

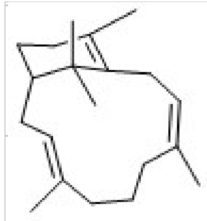
Sobralene

MJP 18-1752-500 in benzene-d6
1H NMR run on 500 MHz NMR

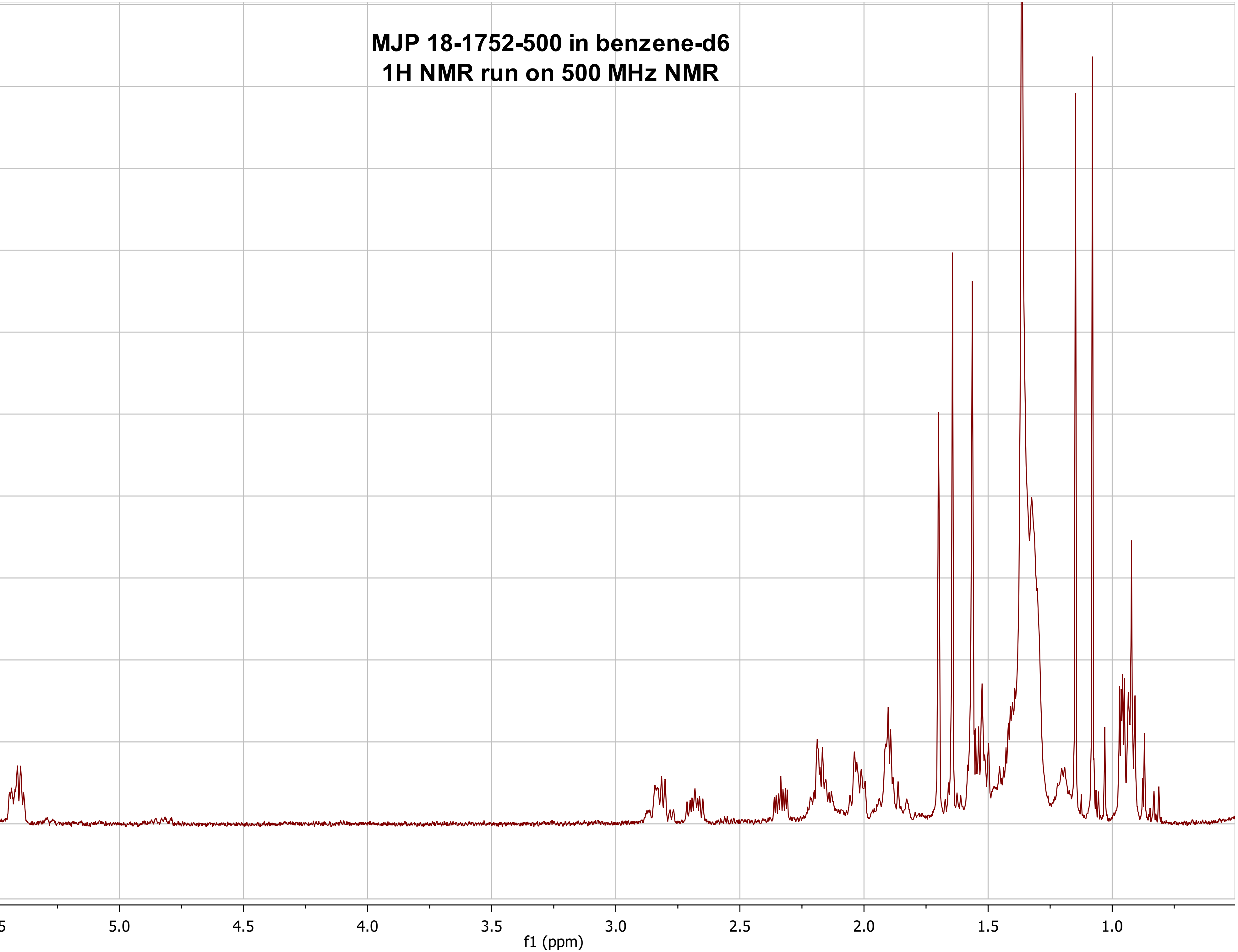

MJP 18-1752-500in benzene-d6
13C NMR run on 500 MHz NMR (126 MHz)

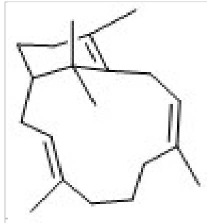

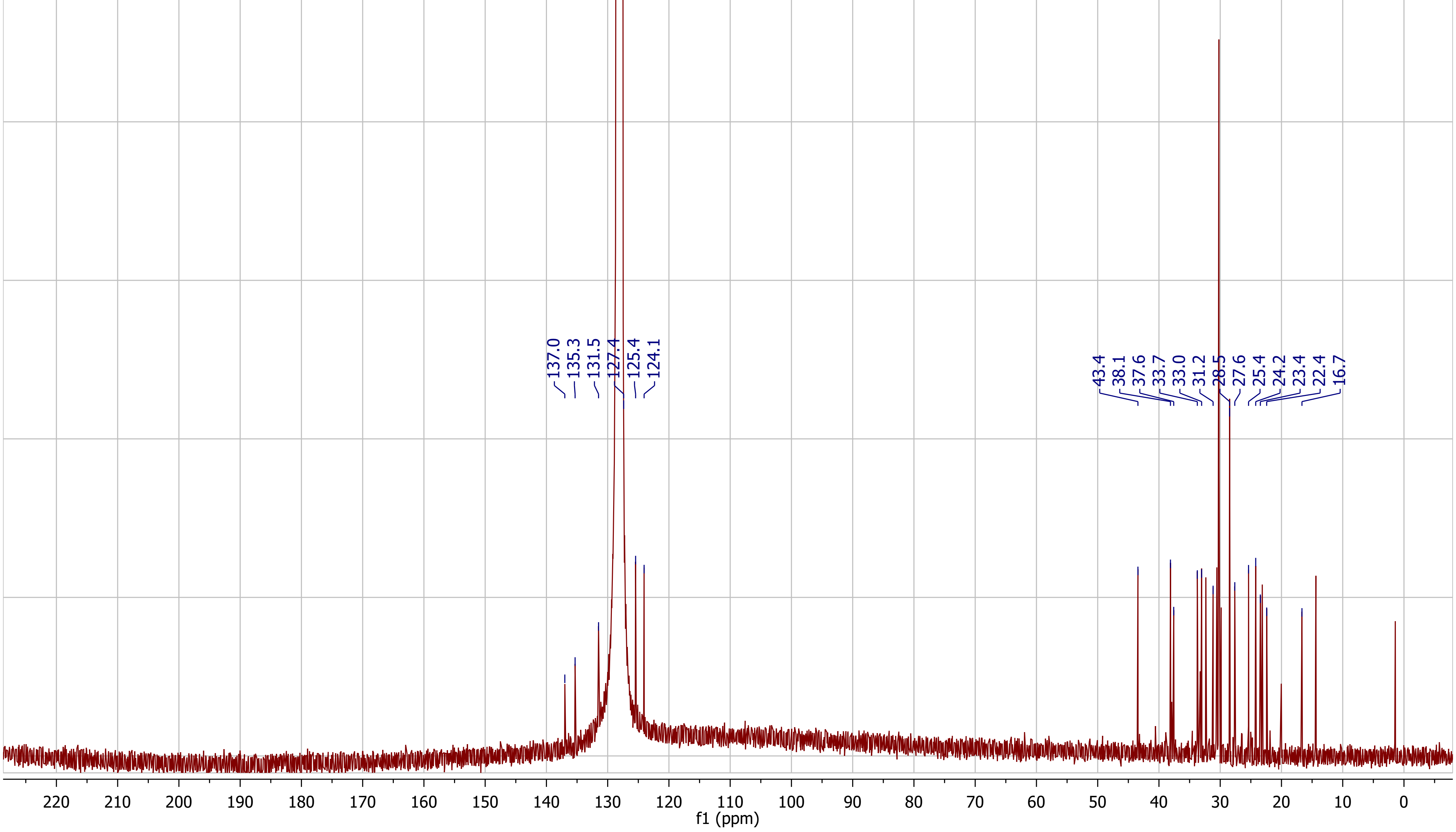

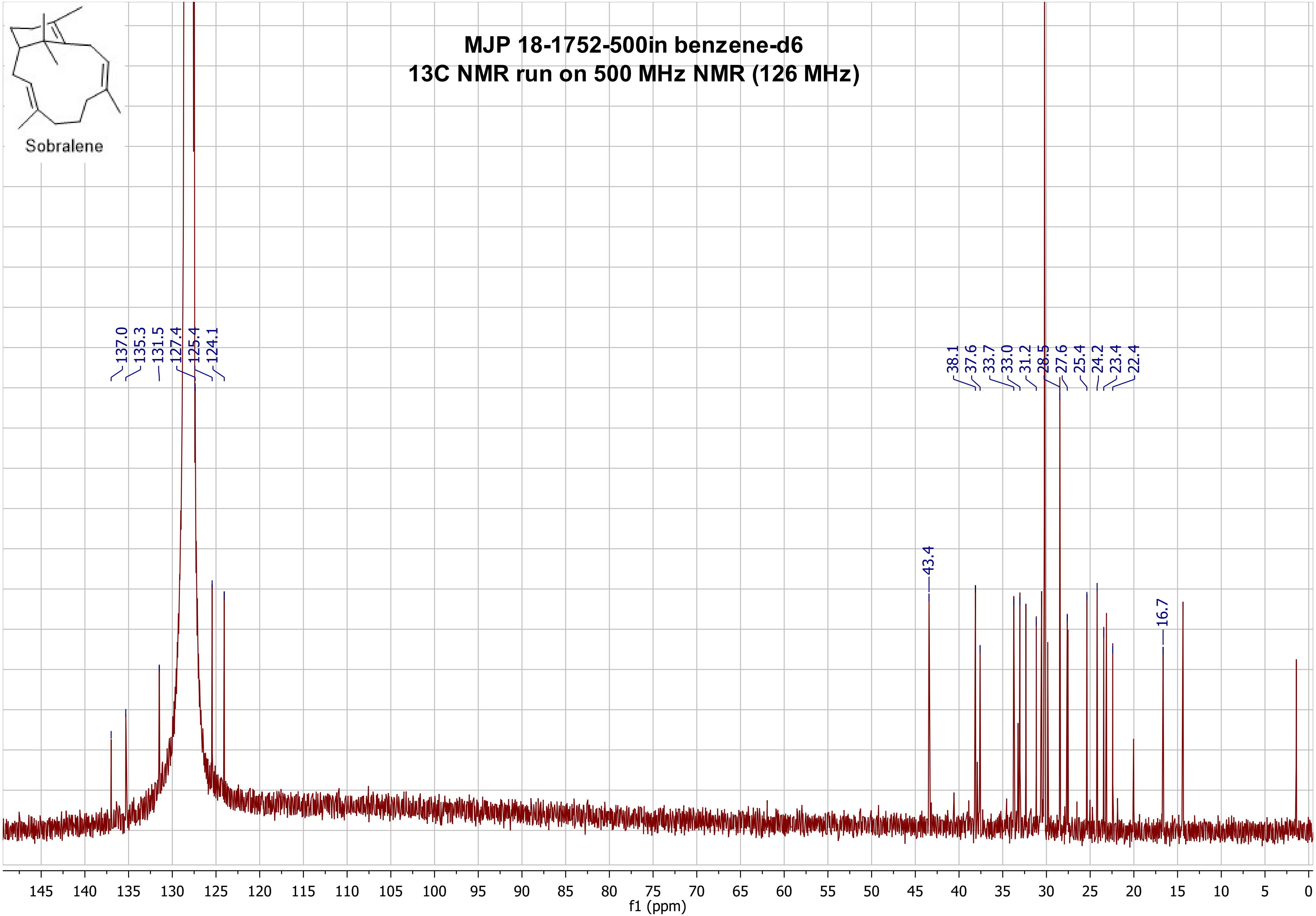
Sobralene
MJP 18-1752-500in benzene-d6
13C NMR run on 500 MHz NMR (126 MHz)
137.0
135.3
131.5
127.4
125.4
124.1
43.4
38.1
37.6
33.7
33.0
31.2
28.5
27.6
25.4
24.2
23.4
22.4
16.7
145 140 135 130 125 120 115 110 105 100 95 90 85 80 75 70 65 60 55 50 45 40 35 30 25 20 15 10 5 0
f1 (ppm)

MJP 18-1752-500 in benzene-d6
COSY NMR run on 500 MHz NMR

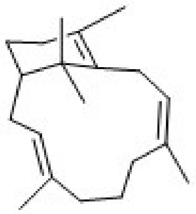

Sobralene

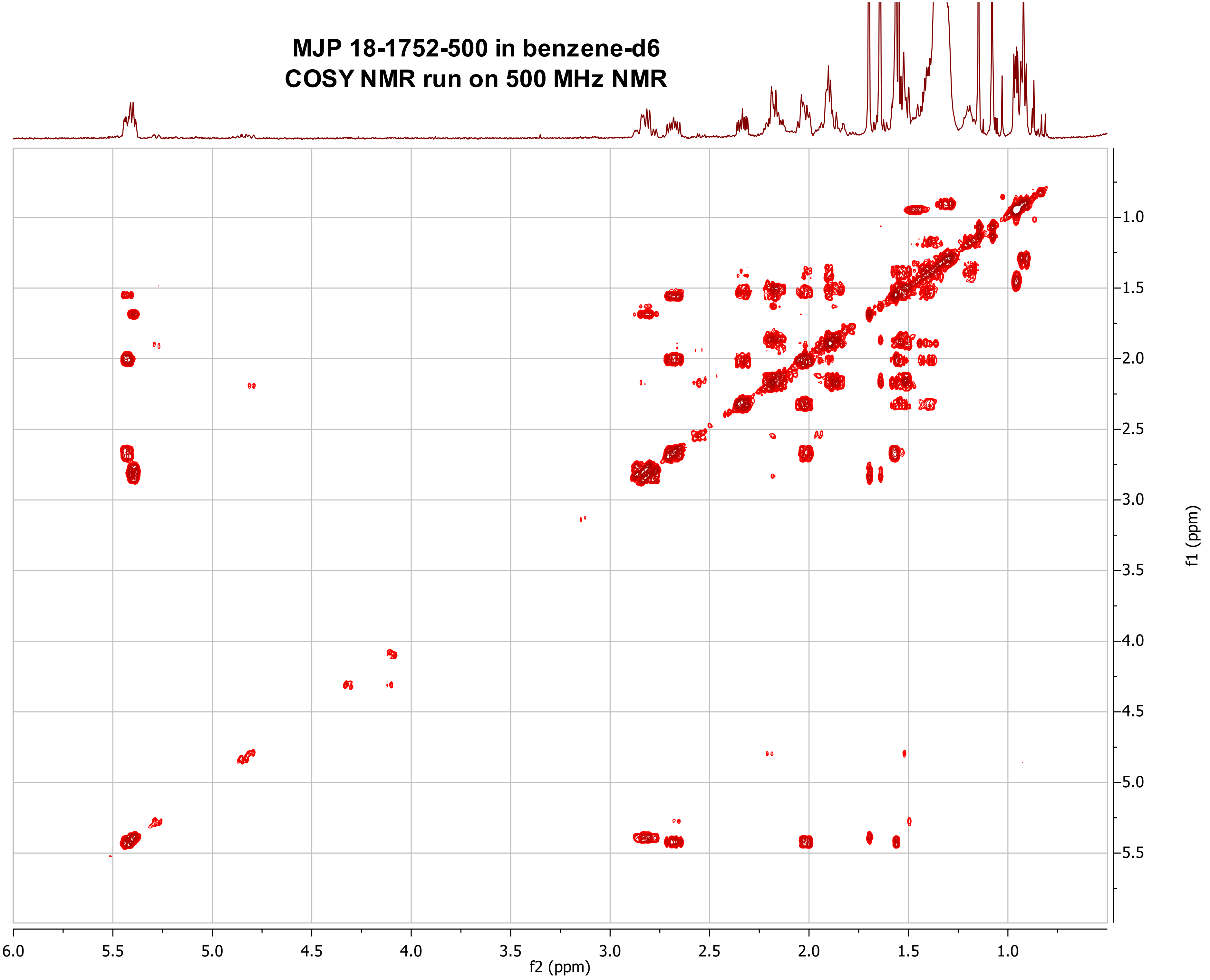

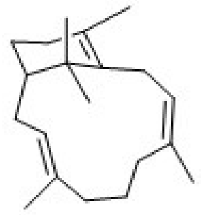

Sobralene

MJP 18-1752-500in benzene-d6
HSQC NMR run on 500 MHz NMR

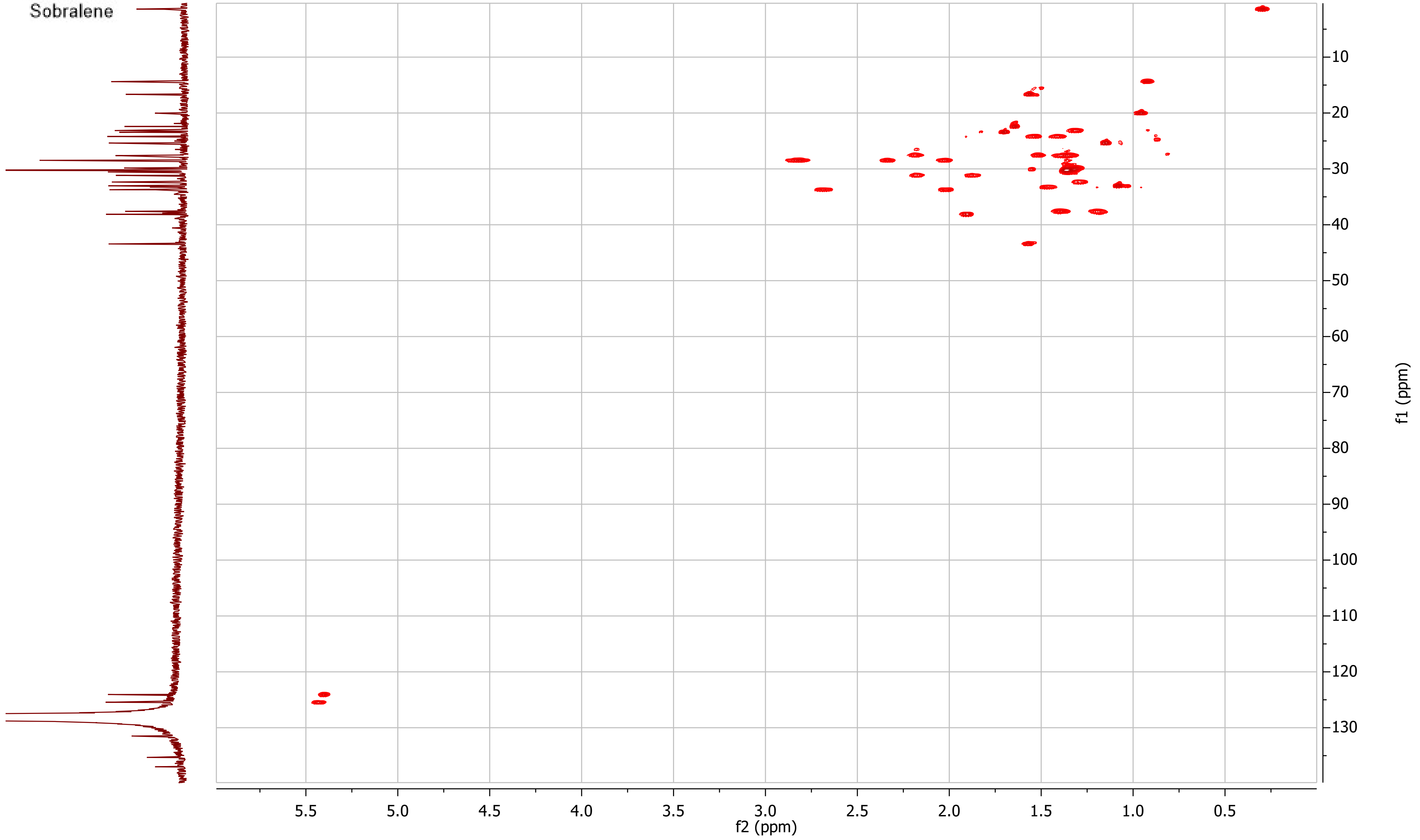

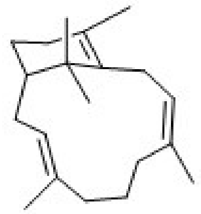

Sobralene

MJP 18-1752-500in benzene-d6
Dept editted HSQC NMR run on 500 MHz NMR

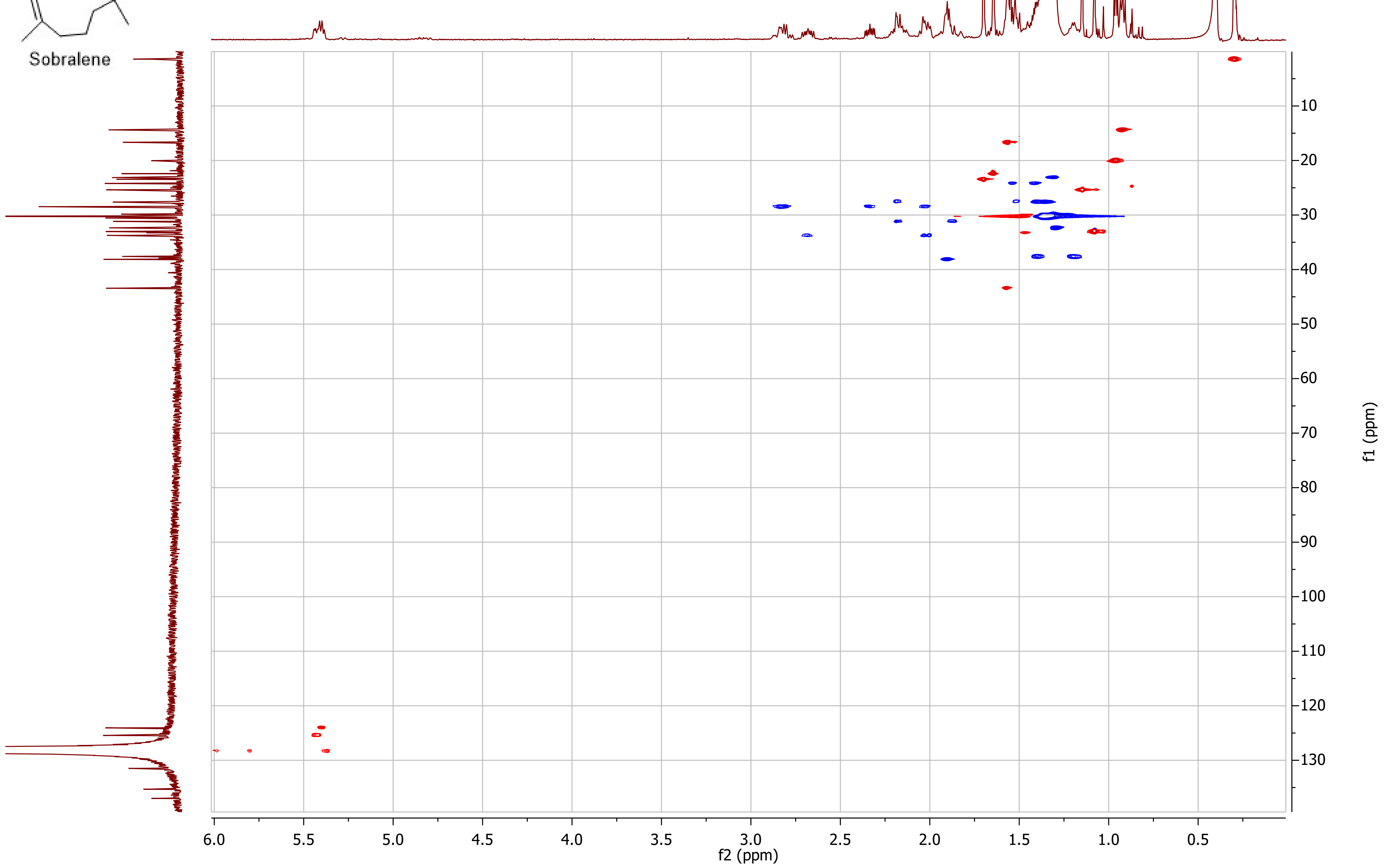

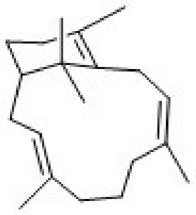

Sobralene

MJP 18-1752-500 in benzene-d6
HMBC NMR run on 500 MHz NMR

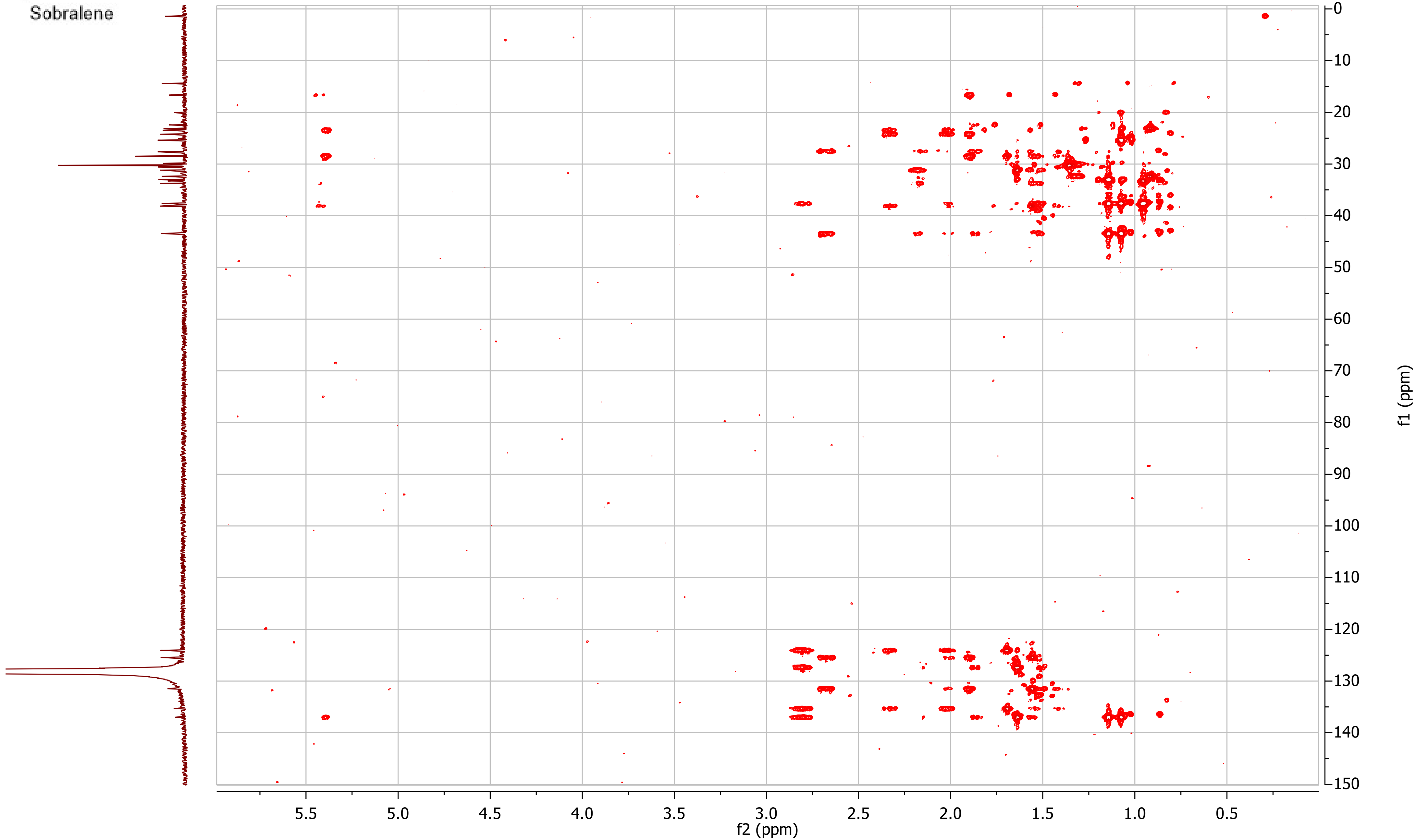

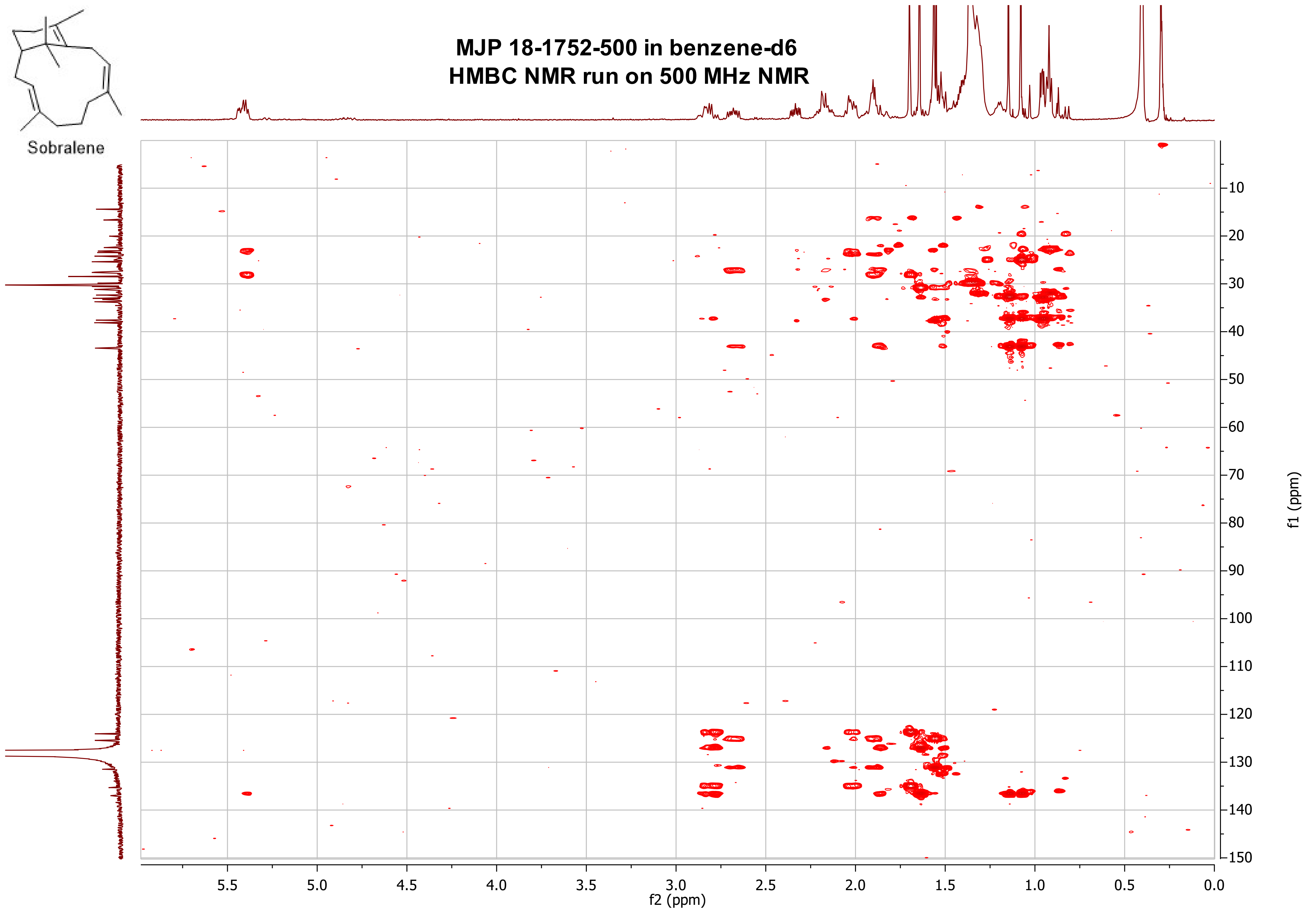

MJP 18-1752-500 in benzene-d6
HMBC NMR run on 500 MHz NMR
Sobralene
f2 (ppm)
f1 (ppm)
5.5
5.0
4.5
4.0
3.5
3.0
2.5
2.0
1.5
1.0
0.5
0.0
10
20
30
40
50
60
70
80
90
100
110
120
130
140
150

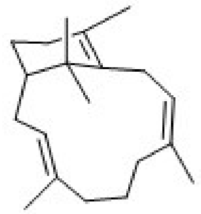

Sobralene

MJP 18-1752-500 in benzene-d6
HMBC NMR run on 500 MHz NMR

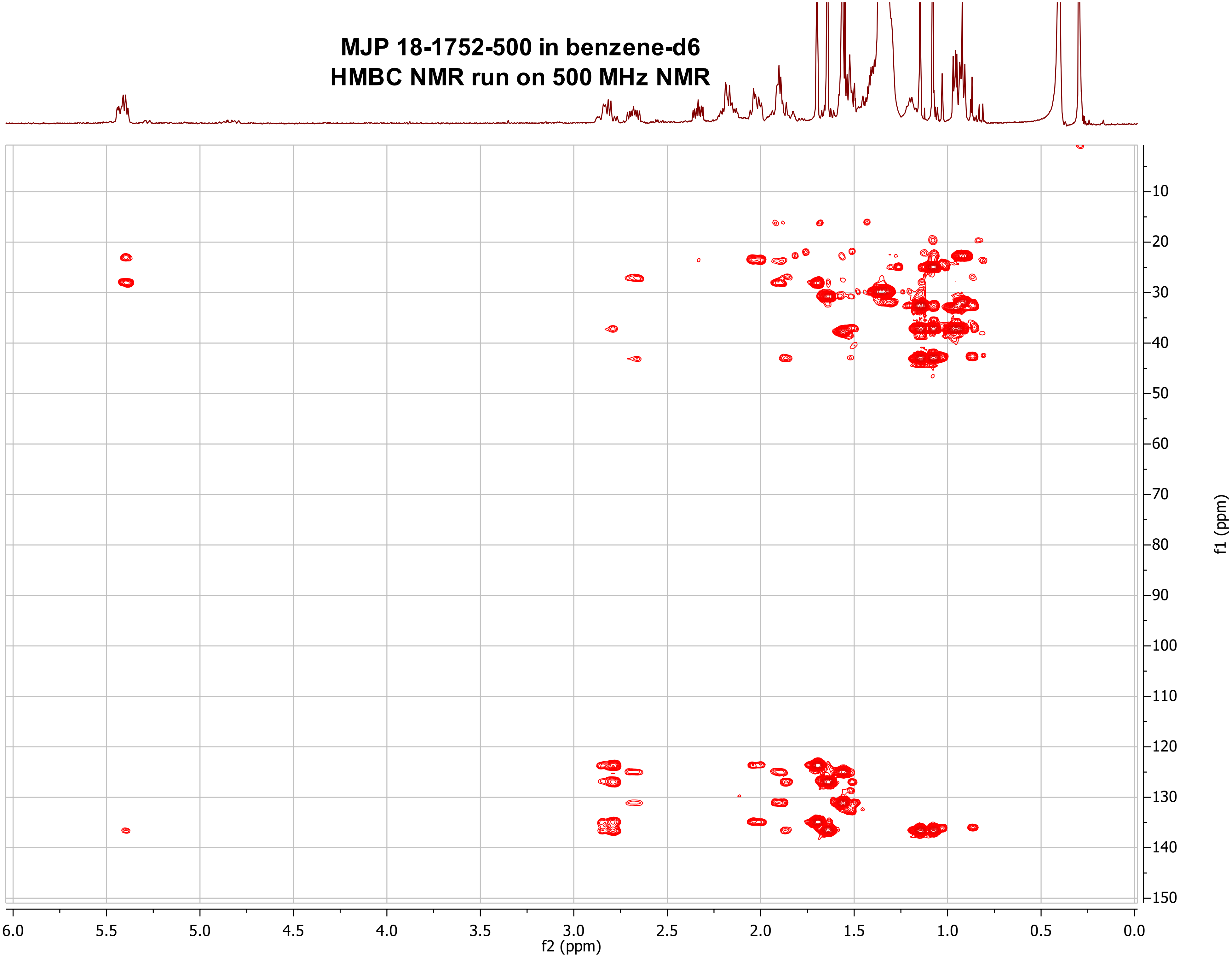

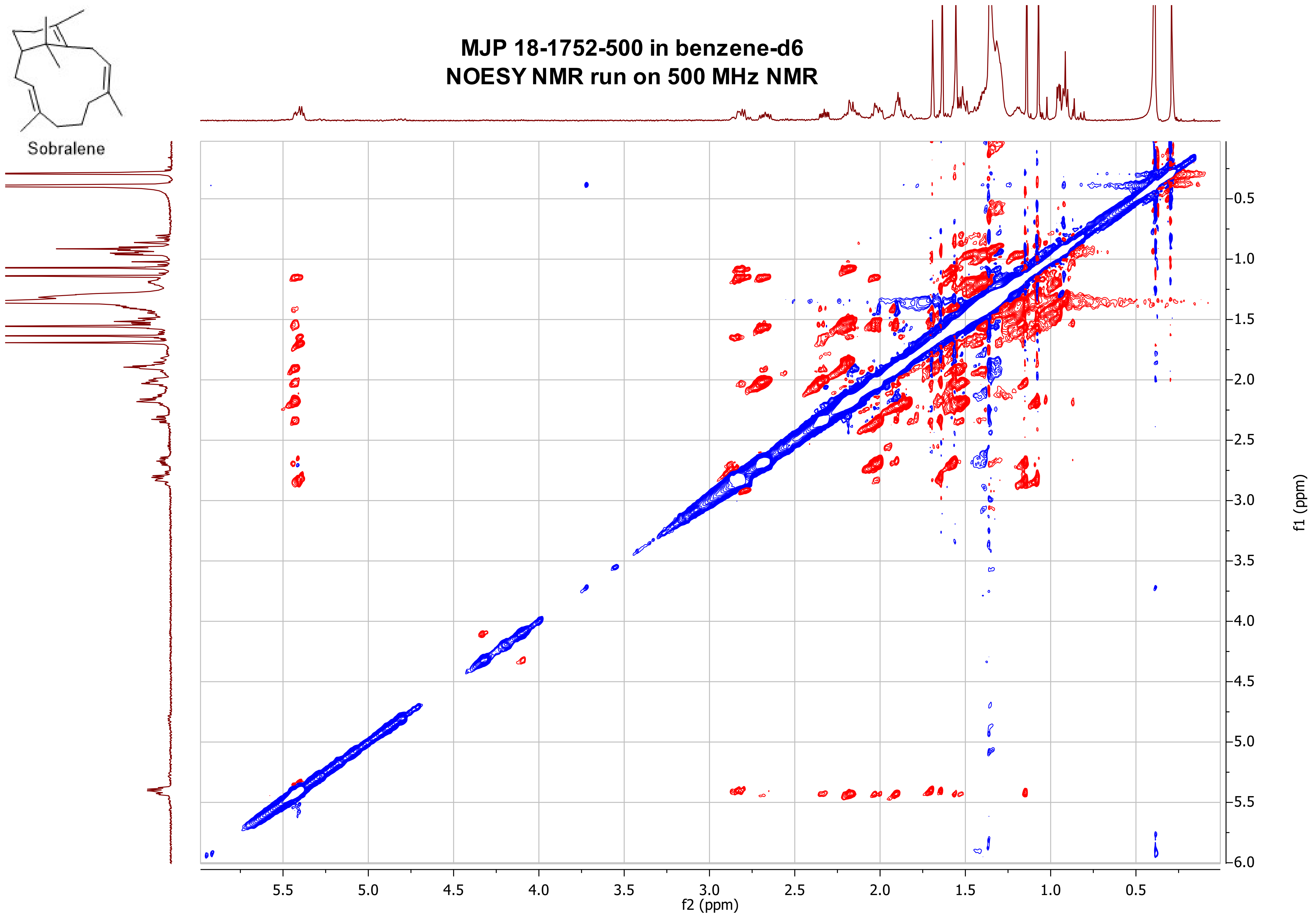
MJP 18-1752-500 in benzene-d6
NOESY NMR run on 500 MHz NMR
Sobralene
f2 (ppm)
f1 (ppm)
5.5
5.0
4.5
4.0
3.5
3.0
2.5
2.0
1.5
1.0
0.5
6.0

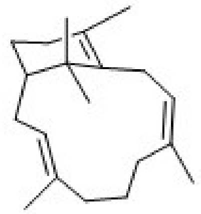

Sobralene

MJP 18-1752-500 in benzene-d6
NOESY NMR run on 500 MHz NMR

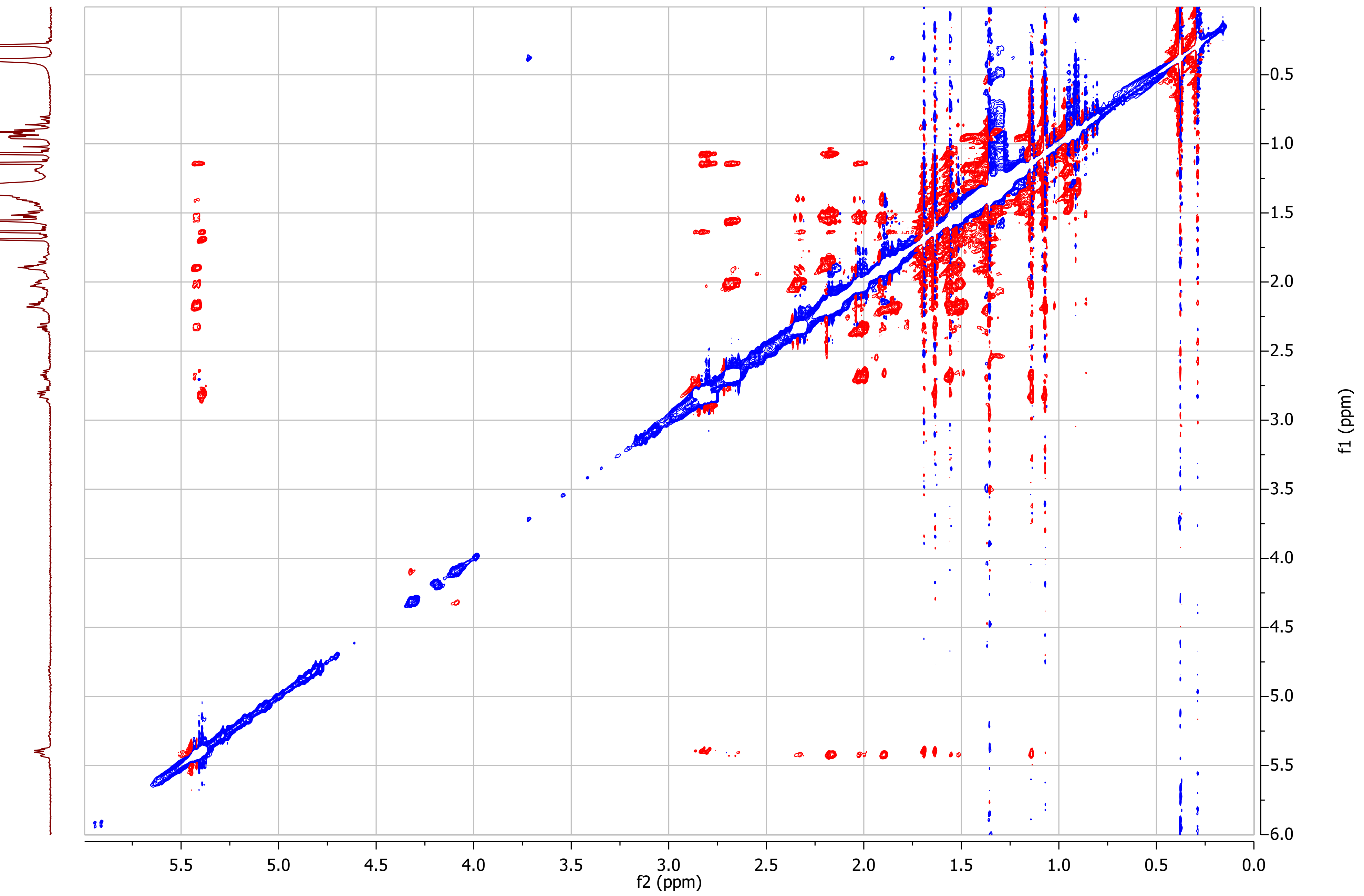

GCMS of Sobralene and Taxadiene

GCMS of sample of Sobralene (expansion of 25 -35 min)

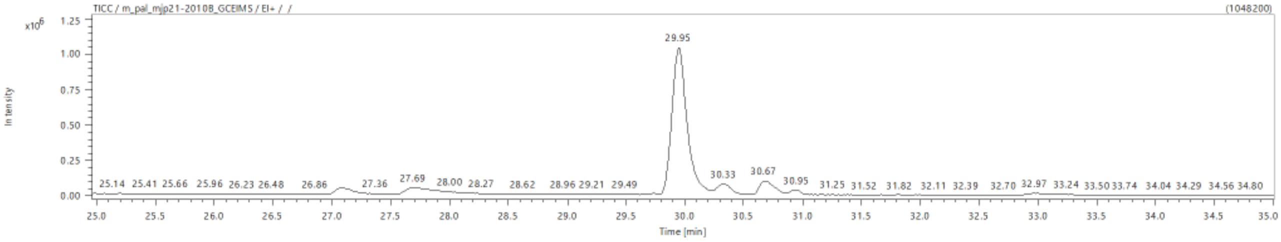

GCMS of sample of Taxadiene (expansion of 25 -35 min)

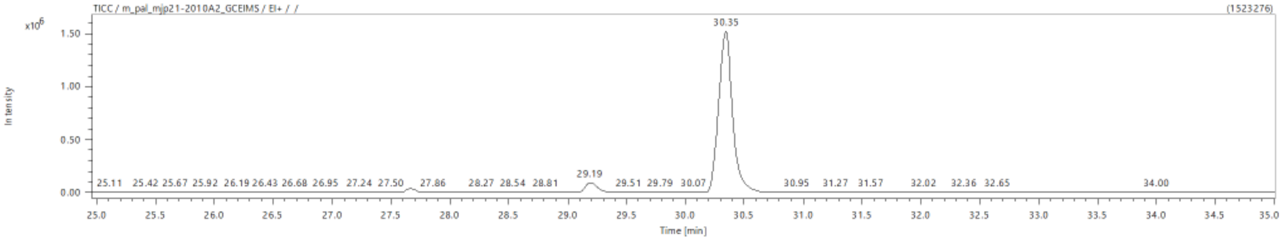

GCMS of sample of Sobralene spiked with Taxadiene (ca 1:1) (expansion of 25 -35 min)

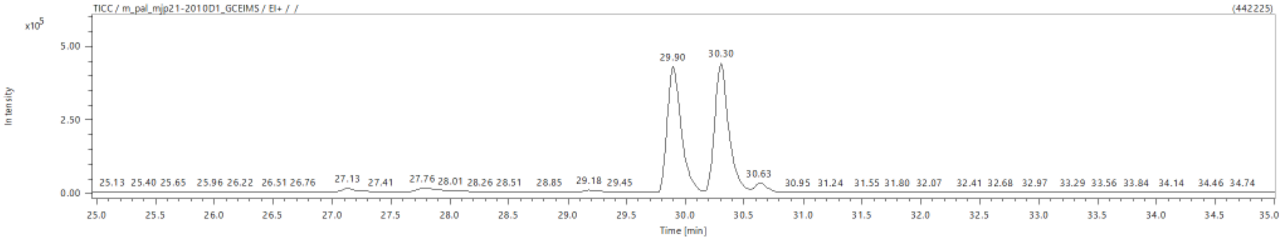

GCMS of sample of Sobralene spiked with Taxadiene (ca 9:1) (expansion of 25 -35 min)

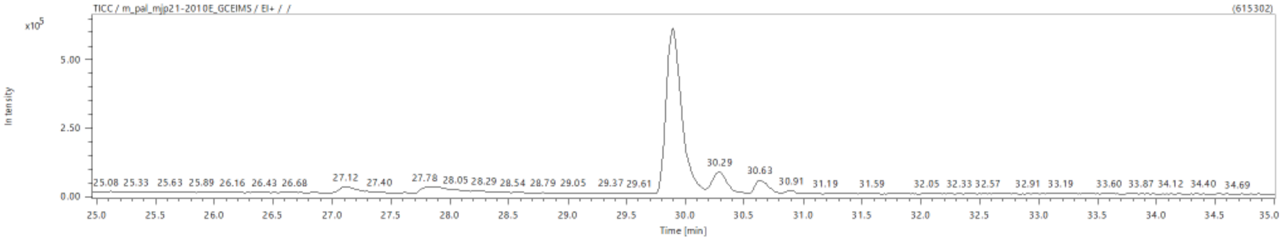

GCMS – Mass spec data for minor peak at (30.30 min)

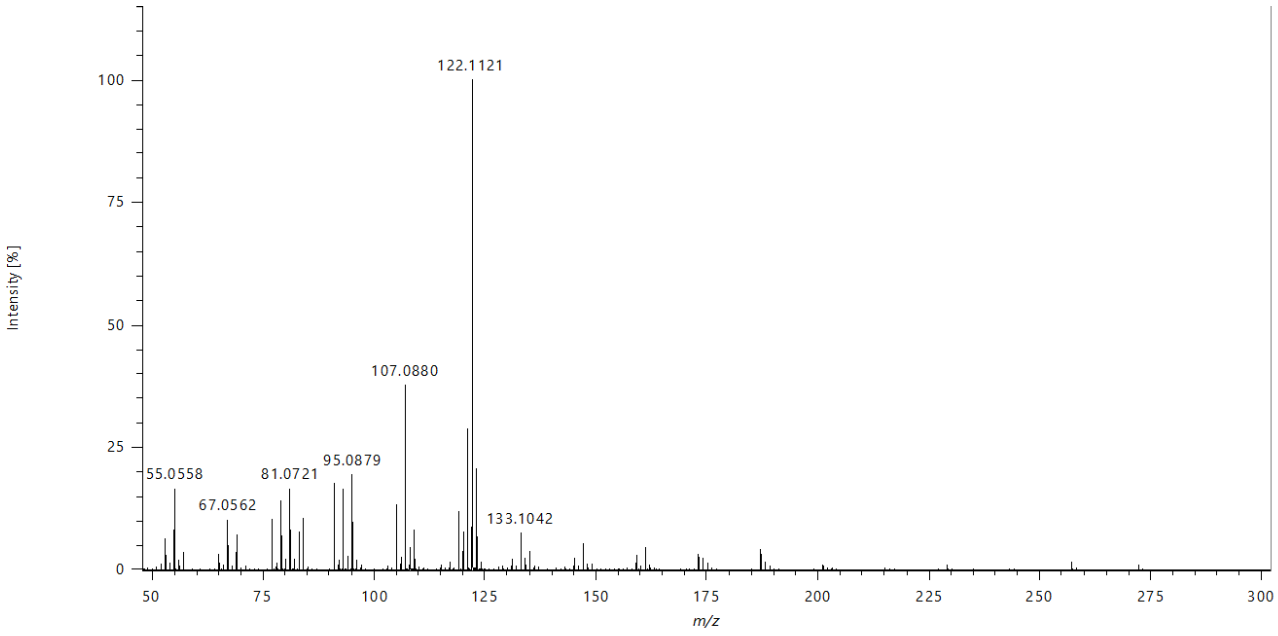

GCMS – Mass spec of Taxadiene (30.30 min)

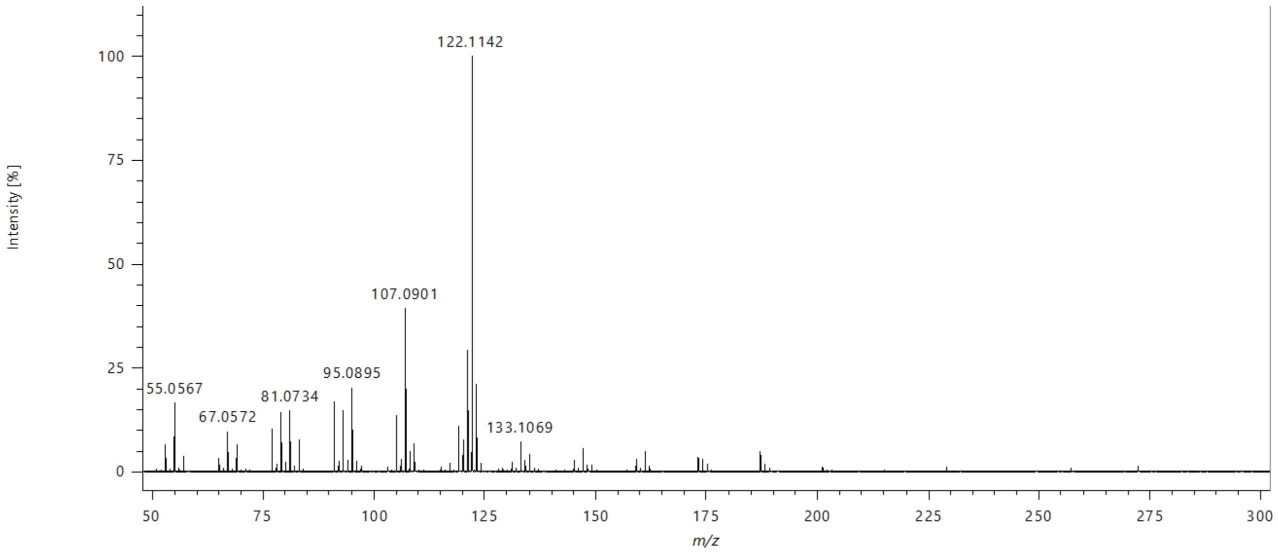

Supplement: Supplementary data 1 [file mmc1.pdf]
